# PIP_2_ depletion promotes TRPV4 channel activity in mouse brain capillary endothelial cells

**DOI:** 10.7554/eLife.38689

**Published:** 2018-08-07

**Authors:** Osama F Harraz, Thomas A Longden, David Hill-Eubanks, Mark T Nelson

**Affiliations:** 1 Department of PharmacologyUniversity of VermontBurlingtonUnited States; 2 Institute of Cardiovascular SciencesManchesterUnited Kingdom; Semmelweis UniversityHungary; The University of Texas at AustinUnited States

**Keywords:** TRPV4, neurovascular coupling, PIP_2_, Kir2.1, GPCR, brain capillaries, phosphoinositides, G_q_PCR, Mouse

## Abstract

We recently reported that the inward-rectifier Kir2.1 channel in brain capillary endothelial cells (cECs) plays a major role in neurovascular coupling (NVC) by mediating a neuronal activity-dependent, propagating vasodilatory (hyperpolarizing) signal. We further demonstrated that Kir2.1 activity is suppressed by depletion of plasma membrane phosphatidylinositol 4,5-bisphosphate (PIP_2_). Whether cECs express depolarizing channels that intersect with Kir2.1-mediated signaling remains unknown. Here, we report that Ca^2+^/Na^+^-permeable TRPV4 (transient receptor potential vanilloid 4) channels are expressed in cECs and are tonically inhibited by PIP_2_. We further demonstrate that depletion of PIP_2_ by agonists, including putative NVC mediators, that promote PIP_2_ hydrolysis by signaling through G_q_-protein-coupled receptors (G_q_PCRs) caused simultaneous disinhibition of TRPV4 channels and suppression of Kir2.1 channels. These findings collectively support the concept that G_q_PCR activation functions as a molecular switch to favor capillary TRPV4 activity over Kir2.1 signaling, an observation with potentially profound significance for the control of cerebral blood flow.

## eLife digest

Capillaries form branching networks that surround all cells of the body. They allow oxygen and nutrient exchange between blood and tissue, but this is not their only role. Capillaries in the brain form a tight barrier that prevents components carried in the blood from easily reaching the brain compartment. They also detect the activity of neurons and trigger on-demand increases in blood flow to active regions of the brain. This role, revealed only recently, depends upon ion channels on the surface of the capillary cells. Active neurons release potassium ions, which open a type of ion channel called Kir2.1 that allows potassium inside the cell to flow out. This process is repeated in neighboring capillary cells until it reaches an upstream vessel, where it causes the vessel to relax and increase the blood flow.

Kir2.1 channels sit astride the membranes of capillary cells, where they can interact with other membrane molecules. One such molecule, called PIP_2_, plays several roles in relaying signals from the outside to the inside of cells. It also physically interacts with channels in the membrane, including Kir2.1 channels. If PIP_2_ levels are low, Kir2.1 channel activity decreases.

Here, Harraz et al. discovered that capillary cells contain another type of ion channel, called TRPV4, which is also regulated by PIP_2_. But unlike Kir2.1, its activity increases when PIP_2_ levels drop. Moreover, TRPV4 channels allow sodium and calcium ions to flow into the cell, which has an effect opposite to that of potassium flowing out of the cell.

Capillary cells also have receptor proteins called G_q_PCRs that are activated by chemical signals released by active neurons in the brain. G_q_PCRs break down PIP_2_, so their activity turns Kir2.1 channels off and TRPV4 channels on. This resets the system so that it is ready to respond to new signals from active neurons. G_q_PCRs work as molecular switches to control the balance between Kir2.1 and TRPV4 channels and turn brain blood flow up and down.

G_q_PCRs and ion channels that depend on PIP_2_ can also be found in other types of cells. These findings could reveal clues about how signals are switched on and off in different cells. Understanding the role of PIP_2_ in signaling could also unveil what happens when signaling go wrong.

## Introduction

Endothelial cells (ECs) line the lumen of all blood vessels and are important regulators of artery and arteriole smooth muscle contractility. In capillaries, which lack an overlying smooth muscle cell layer, this regulatory function is absent. In these smallest of all blood vessels, ECs instead support the nutrient- and gas-exchange function characteristic of capillary beds generally. In the brain, capillary ECs (cECs) also constitute the blood-brain barrier, reflecting the presence of tight junctions between adjacent cECs. Not surprisingly given these differing cellular missions, the molecular repertoire of brain cECs and arterial/arteriolar ECs exhibit some marked differences. Notably, intermediate and small-conductance Ca^2+^-activated K^+^ channels (IK and SK, respectively), which are important in transducing elevations in intracellular Ca^2+^ concentration ([Ca^2+^]_i_) into membrane potential hyperpolarization in artery/arteriolar ECs to cause relaxation of electrically coupled smooth muscle cells (Ledoux et al., 2008; Sonkusare et al., 2012; Taylor et al., 2003), are absent in brain cECs (Longden et al., 2017). However, signaling through G protein–coupled receptors of the Gα_q/11_-subtype (G_q_PCRs)—an important component of this smooth muscle regulatory axis—is robust in brain cECs (Harraz et al., 2018). In the canonical pathway of G_q_PCR signaling, Gα_q_ released upon agonist binding activates phospholipase C (PLC), which hydrolyzes the minor inner leaflet phospholipid, phosphatidylinositol 4,5-bisphosphate (PIP_2_). Breakdown of PIP_2_ yields diacylglycerol (DAG) and inositol 1,4,5-trisphosphate (IP_3_), the latter of which increases [Ca^2+^]_i_ by acting on its cognate receptor (IP_3_R) on the endoplasmic reticulum (ER) to promote Ca^2+^ release from intracellular stores.

Blood delivery within the brain is mediated by an ever-narrowing vascular tree comprising surface arteries, penetrating (parenchymal) arterioles and a vast network of capillaries, which enormously extend the territory of perfusion (Blinder et al., 2013). Because the brain lacks substantial energy reserves, it relies on an on-demand mechanism for redistributing oxygen and nutrients to regions of higher neuronal activity, a process in which products released by active neurons trigger a local increase in blood flow. This use-dependent increase in local blood flow (functional hyperemia), mediated by a process termed neurovascular coupling (NVC), is essential for normal brain function (Iadecola and Nedergaard, 2007) and represents the physiological basis for functional magnetic resonance imaging (Raichle and Mintun, 2006).

We recently reported that brain capillaries act as a neuronal activity-sensing network, demonstrating that brain cECs are capable of initiating an electrical (hyperpolarizing) signal in response to neuronal activity that rapidly propagates upstream to dilate feeding parenchymal arterioles and increase blood flow locally at the site of signal initiation (Longden et al., 2017). We also established the molecular mechanism underlying this process, showing that extracellular K^+^—a byproduct of every neuronal action potential—is the critical mediator and identifying the Kir2.1 channel as the key molecular player (Longden et al., 2017). Kir2.1 channels are activated by external K^+^ and exhibit steep activation by hyperpolarization (Hibino et al., 2010; Longden and Nelson, 2015), characteristics that facilitate regenerative K^+^ conductance in neighboring cECs and enable long-range propagation of the hyperpolarizing signal (Longden et al., 2017). Intriguingly, we have since discovered that decreases in capillary endothelial PIP_2_ levels induced by G_q_PCRs agonists suppress Kir2.1 channel currents, reflecting the fact that PIP_2_ is required for Kir2.1 channel activity (Harraz et al., 2018). Importantly, this suppressive effect of PIP_2_ depletion exerts a major modulatory influence on K^+^-evoked hyperemic responses in vivo. In addition to regulating Kir2 family members, PIP_2_ has been shown to bind to and regulate a diverse array of ion channels (Hille et al., 2015). Notable in this context, PIP_2_ binding has been shown to inhibit several members of the transient receptor potential (TRP) family of non-selective cation channels, including the vanilloid subtype TRPV4 (Nilius et al., 2008; Rohacs, 2009; Takahashi et al., 2014), which we and others have shown is an important Ca^2+^ influx pathway in arterial and arteriolar ECs (Earley and Brayden, 2015; Sonkusare et al., 2014;Sonkusare et al., 2012; White et al., 2016).

The Kir2.1 channel appears to be the major K^+^ channel type in brain cECs (Longden et al., 2017). However, the identity of depolarizing (Na^+^/Ca^2+^-permeable) channels in cECs is not known. Here, we show that TRPV4 channels are present in cECs and exhibit an exceedingly low open probability under basal conditions due to tonic inhibition by PIP_2_. We have further found that this inhibition is relieved through PIP_2_ depletion, independent of the action of the PIP_2_ hydrolysis products, DAG and IP_3_. These findings provide a counterpoint to our recent demonstration that Kir2.1 channel activity is sustained by basal levels of PIP_2_ and suppressed by G_q_PCR-mediated PIP_2_ depletion (Harraz et al., 2018). Collectively, our findings indicate that PIP_2_ exerts opposite effects on TRPV4 and Kir2.1 channels in brain cECs, demonstrating that a single regulatory pathway governs the balance between two divergent signaling modalities—one depolarizing and the other hyperpolarizing—in the capillary endothelium.

## Results

### TRPV4 channels in brain cECs are inhibited by intracellular ATP

**Figure 1.**
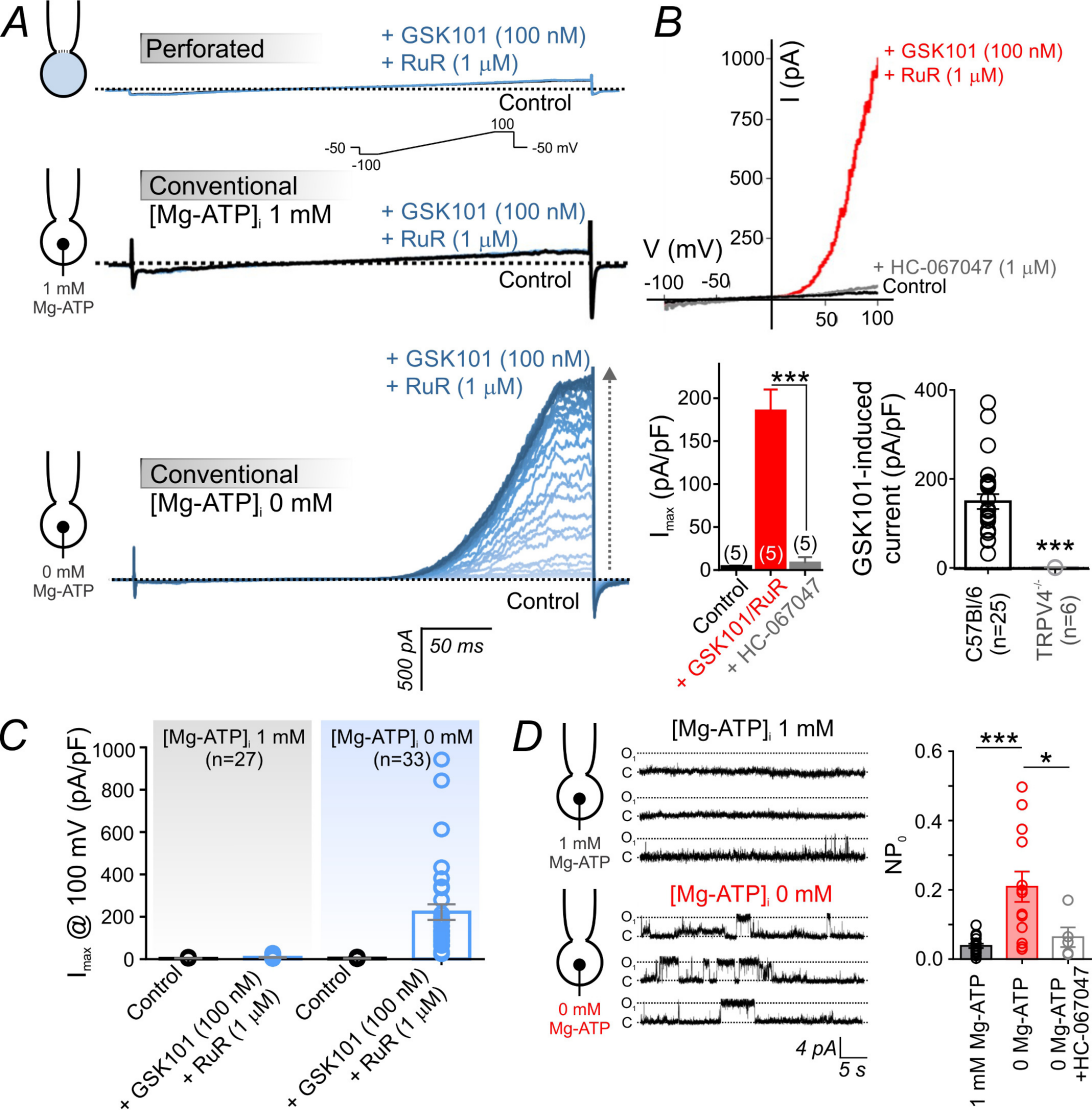
Intracellular ATP suppresses TRPV4 currents in cECs. (**A**) Representative traces of TRPV4 currents recorded from freshly isolated cECs using voltage ramps (−100 to 100 mV, from a holding potential −50 mV; inset) and different patch-clamp configurations before (black) and after (blue) the application of 100 nM GSK101 and 1 µM RuR. *Top*: Currents recorded in the perforated whole-cell configuration. *Middle*: Currents recorded in the conventional whole-cell configuration (dialyzed cytoplasm, 1 mM Mg-ATP in the pipette solution). *Bottom*: Currents recorded in the conventional whole-cell configuration (0 mM Mg-ATP in the pipette solution) developed gradually over ~4 min. (**B**) Current-voltage relationship (*top*) and summary data (*bottom left*) of currents recorded before (control) and after the cumulative application of GSK101 (100 nM)+RuR (1 µM) followed by HC-067047 (1 µM) (means ± SEM, ***p<0.001, unpaired Student’s t-test; n = 5 each). *Bottom right*: Individual-value plot of peak outward GSK101 (100 nM)-induced currents in cECs isolated from brains of C57Bl/6 (n = 25) or TRPV4^-/-^ (n = 6) mice. A minimum duration of ~5 min after the application of GSK101 was allowed for outward TRPV4 current to develop in each cEC. Data are presented as means ± SEM (***p<0.001, unpaired Student’s t-test). (**C**) Individual-value plot of peak outward currents recorded at 100 mV before and after the application of GSK101 (100 nM) onto cECs dialyzed with 0 or 1 mM Mg-ATP in the pipette solution. Individual data points are shown together with means (column bars) and SEM (error bars). (**D**) Representative traces (*left*) and summary individual-value plot (*right*) of TRPV4 single-channel activity. Single-channel openings of TRPV4 channels were recorded as outward quantal K^+^ currents from cECs in the absence of GSK101 (conventional whole-cell configuration; holding potential, +50 mV; sampling rate, 20 kHz; low-pass filter frequency, 1 kHz; average recording time for each data point, 6 min). cECs were dialyzed with 0 mM (n = 13) or 1 mM (n = 16) Mg-ATP. One group of cECs dialyzed with 0 mM Mg-ATP was treated with 1 µM HC-067047 (n = 5). Data are presented as means (column bars) ± SEM (error bars; *p<0.05, ***p<0.001, one-way ANOVA followed by Tukey’s multiple comparisons test). 10.7554/eLife.38689.007Figure 1—source data 1.Numerical data that were used to generate the chart in Figure 1B. 10.7554/eLife.38689.008Figure 1—source data 2.Numerical data that were used to generate the chart in Figure 1C. 10.7554/eLife.38689.009Figure 1—source data 3.Numerical data that were used to generate the chart in Figure 1D.

We previously reported that the selective TRPV4 agonist GSK1016790A (hereafter GSK101) is a potent activator of TRPV4 currents in mesenteric artery ECs that induces maximal arterial dilation at concentrations in the low nanomolar range (Sonkusare et al., 2014; Sonkusare et al., 2012). To assess TRPV4 channel expression and function in brain cECs, we first performed patch-clamp electrophysiology experiments on freshly isolated cECs from C57BL/6J mouse brain slices, prepared as described in Materials and methods. Specifically, we recorded outward K^+^ currents mediated by TRPV4 channels in the cytoplasm-intact, perforated whole-cell configuration using a 300 ms ramp protocol (−100 to +100 mV, from a holding potential of −50 mV). Recordings were made in the presence of the voltage-dependent pore blocker ruthenium red (RuR; 1 µM), an approach we have previously used to assess TRPV4 currents in mesenteric artery ECs (Sonkusare et al., 2012). Under these conditions, depolarizing ramps rapidly displace RuR, allowing unimpeded outward K^+^ currents to be detected while preventing Na^+^/Ca^2+^ influx and associated Ca^2+^ overload and cell death (Sonkusare et al., 2012). Unexpectedly, we found that GSK101 was unable to evoke a detectable whole-cell TRPV4 current in cECs at concentrations that maximally activate TRPV4 channels in mesenteric artery ECs; in fact, even GSK101 concentrations as high as 100 nM failed to stimulate a detectable TRPV4 current in isolated cECs (Figure 1A). However, we serendipitously found that breaking through the cell membrane from a perforated patch in the presence of an otherwise ineffective GSK101 concentration (100 nM) resulted in the gradual (~5 min) development of a robust TRPV4 current, suggesting that a factor that suppresses TRPV4 channels had been washed out of the cell.

**Figure 1—figure supplement 1.**
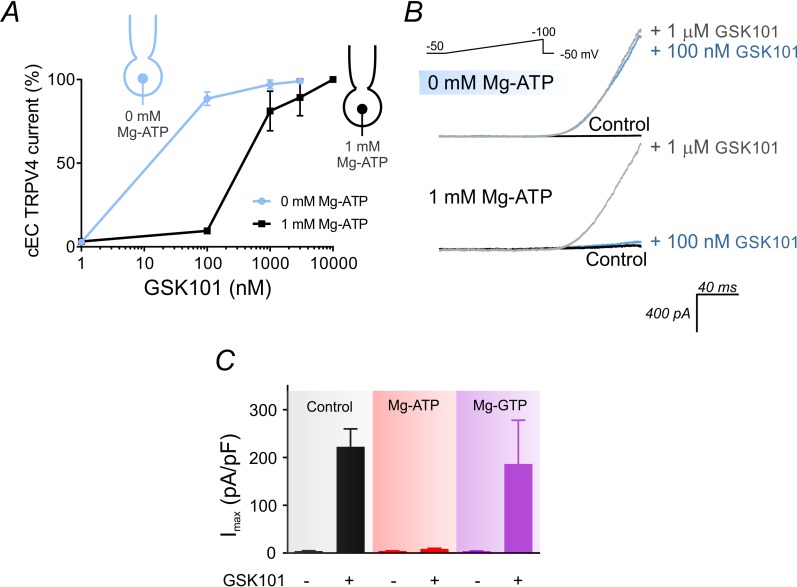
Intracellular ATP suppresses TRPV4 activity in cECs. (**A**) GSK101 concentration-response curve. Currents were measured at 100 mV using the conventional whole-cell configuration in cECs in the presence of 1 µM RuR. cECs were dialyzed with 0 or 1 mM Mg-ATP in the pipette solution and GSK101-evoked currents developed over ~5 min. TRPV4 current densities with 100 nM GSK101 were 192 ± 30 pA/pF (0 mM Mg-ATP) and 13 ± 2 pA/pF (1 mM Mg-ATP). Data are presented as means ± SEM (0–1000 nM GSK101, n = 9 cECs; 3–10 µM GSK101, n = 2–3 cECs). (**B**) Representative traces from two cECs dialyzed with 0 mM Mg-ATP (*upper*) or 1 mM Mg-ATP (*lower*). Currents were recorded using voltage ramps (−50 to 100 mV) before and after the application of 100 nM and then 1 µM GSK101. (**C**) Summary data showing outward currents at 100 mV, before and after 100 nM GSK101, recorded in dialyzed cECs using different intracellular nucleotide compositions: no nucleotides (control), 1 mM Mg-ATP, or 1 mM Mg-GTP (n = 7–25). For each cEC, a minimum of 5 min was allowed for outward TRPV4 current to develop after the application of GSK101.

**Figure 1—figure supplement 2.**
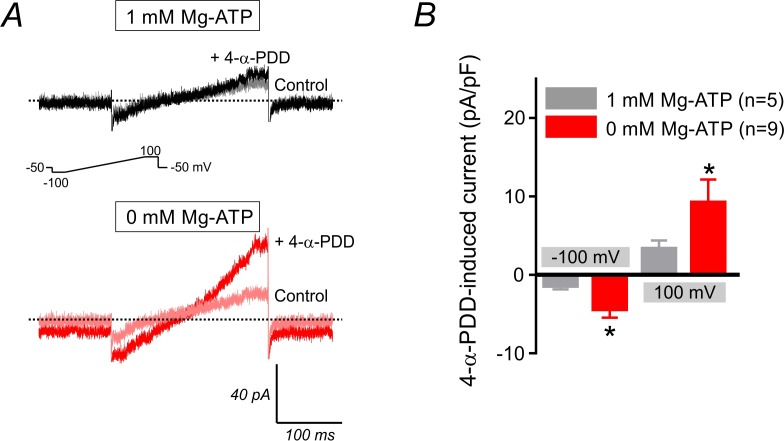
4-α-PDD-induced current in cECs. (**A**) Representative traces recorded from two cECs dialyzed with 1 mM (*upper*) and 0 mM Mg-ATP (*lower*), before and after the application of 4-α-PDD (5 µM), using 300 ms voltage ramps (−100 to 100 mV, inset). Ruthenium red was not used in this experiment. (**B**) Summary data represent 4-α-PDD (5 µM)-induced inward (at −100 mV) and outward (at 100 mV) currents in cECs dialyzed with 0 or 1 mM Mg-ATP. Data represent means ± SEM (*p*<0.05* unpaired Student’s t-test vs. the 1 mM Mg-ATP condition, n = 5–9 cECs per condition).

**Figure 1—figure supplement 3.**
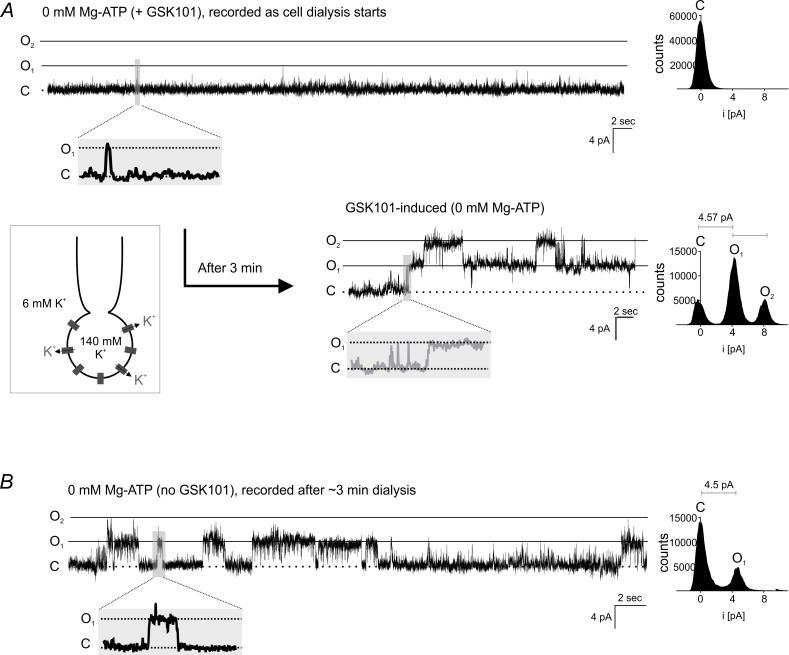
Single-channel TRPV4 currents in cECs. (**A**) Agonist-induced single-cell TRPV4 currents in a cEC. *Top*: Representative trace (*left*) and corresponding histogram (*right*) of single-channel activity in a cEC dialyzed with 0 mM Mg-ATP. Currents were recorded in the conventional whole-cell configuration in the presence of 100 nM GSK101 immediately after gaining electrical access to the cell (holding potential, +50 mV; sampling rate, 20 kHz; low-pass filter frequency, 1 kHz). *Bottom*: Trace and histogram for the same cEC recorded ~3 min after initiating dialysis when the effect of GSK101 commenced. The amplitude histogram revealed a unitary TRPV4 current amplitude (i) of 4.57 pA. (**B**) Single-cell TRPV4 currents in the absence of GSK101. Traces and corresponding histogram of single TRPV4 channel currents in a cEC dialyzed with 0 mM Mg-ATP (conventional whole-cell configuration) in the absence of an agonist. The cEC was held at +50 mV, and currents were recorded after dialyzing for ~3 min. The unitary current amplitude was 4.5 pA.

In the conventional whole-cell configuration with physiological levels of Mg-ATP (1 mM) included in the patch pipette, application of 100 nM GSK101 also failed to evoke an outward current. In striking contrast, this same concentration of GSK101 caused the gradual (~4–5 min) development of a robust outward current in the conventional whole-cell configuration when Mg-ATP was omitted from the pipette solution (Figure 1A). Collectively, these observations suggest that ATP is the suppressive factor in question. Consistent with this interpretation, intracellular Mg-ATP (1 mM) caused a dramatic shift in the sensitivity of cEC TRPV4 channels to GSK101: at a potential of 100 mV, the EC_50_ of GSK101 for TRPV4 channels in the presence of Mg-ATP was 415 nM. However, in the absence of Mg-ATP, the EC_50_ was reduced ~23 fold to 18 nM (Figure 1C; Figure 1—figure supplement 1A,B), a value comparable to that observed in mesenteric artery ECs (Sonkusare et al., 2014). The suppressive effect of ATP was specific for adenosine nucleotides, as inclusion of 1 mM GTP in the patch pipette did not alter currents (Figure 1—figure supplement 1C). GSK101-induced currents were completely abolished by the selective TRPV4 channel antagonist HC-067047 (1 µM) and were absent in cECs from TRPV4-knockout (TRPV4^-/-^) mice (Figure 1B; Figure 1—source data 1), confirming that these currents are attributable to TRPV4 channels. The inhibitory effect of intracellular Mg-ATP (1 mM) was not limited to GSK101-activated TRPV4 currents. As shown (Figure 1—figure supplement 2), it also suppressed both inward and outward currents in cECs activated by the TRPV4 agonist 4α-phorbol 12,13-didecanoate (4α-PDD; 5 µM, in the absence of RuR).

There was no detectable whole-cell TRPV4 current in the absence of a TRPV4 activator (Figure 1A), suggesting that these channels have a very low basal open probability. To further investigate this, we monitored single TRPV4 channel openings using dialyzed cECs in the conventional whole-cell configuration, an approach that should allow detection of openings of all channels throughout the cEC plasma membrane while controlling intracellular composition. First, we measured TRPV4 unitary currents in dialyzed cECs (0 mM Mg-ATP) held at +50 mV before and after the application of 100 nM GSK101. The unitary current amplitude in the presence of GSK101, estimated from amplitude histograms, was 4.6 ± 0.3 pA (n = 3) (Figure 1—figure supplement 3A). This value corresponds to a single-channel conductance of 92 ± 6 pS, in line with values reported in an earlier investigation of TRPV4 channels in native arterial ECs (Watanabe et al., 2002) and heterologous expression systems (Loukin et al., 2010). Extending these results, we next performed a series of single-channel recordings in the absence of GSK101. cECs held at +50 mV and dialyzed with Mg-ATP (1 mM) displayed infrequent openings (NP_O_ = 0.038 ± 0.006), but removing ATP increased single-channel opening by ~6 fold (NP_O_ = 0.209 ± 0.044) (Figure 1D; Figure 1—source data 3). Single-channel openings in the absence of agonist exhibited a unitary current of 4.5 ± 0.3 pA at +50 mV (Figure 1D, Figure 1—figure supplement 3B), similar to that evoked using a specific TRPV4 agonist (4.6 pA) (Figure 1—figure supplement 3A) and corresponding to a conductance of 90 ± 6 pS. Single-channel currents recorded in the absence of GSK101 were inhibited by the TRPV4 blocker HC-067047 (NP_O_ = 0.063 ± 0.028) (Figure 1D; Figure 1—source data 3), confirming their identity as TRPV4 channel-mediated currents. Based on our estimate of ~225 functional TRPV4 channels per cEC (see Materials and methods), we calculate that capillary TRPV4 channels have a basal open probability (P_O_) of ~0.0002. These observations collectively indicate that capillary TRPV4 channels exhibit a low open probability under basal conditions, and that both constitutive and agonist-induced TRPV4 activities are suppressed by intracellular ATP and recover following ATP washout/dialysis.

### TRPV4 channel suppression is dependent on ATP hydrolysis, but independent of protein kinase A, G or C

**Figure 2.**
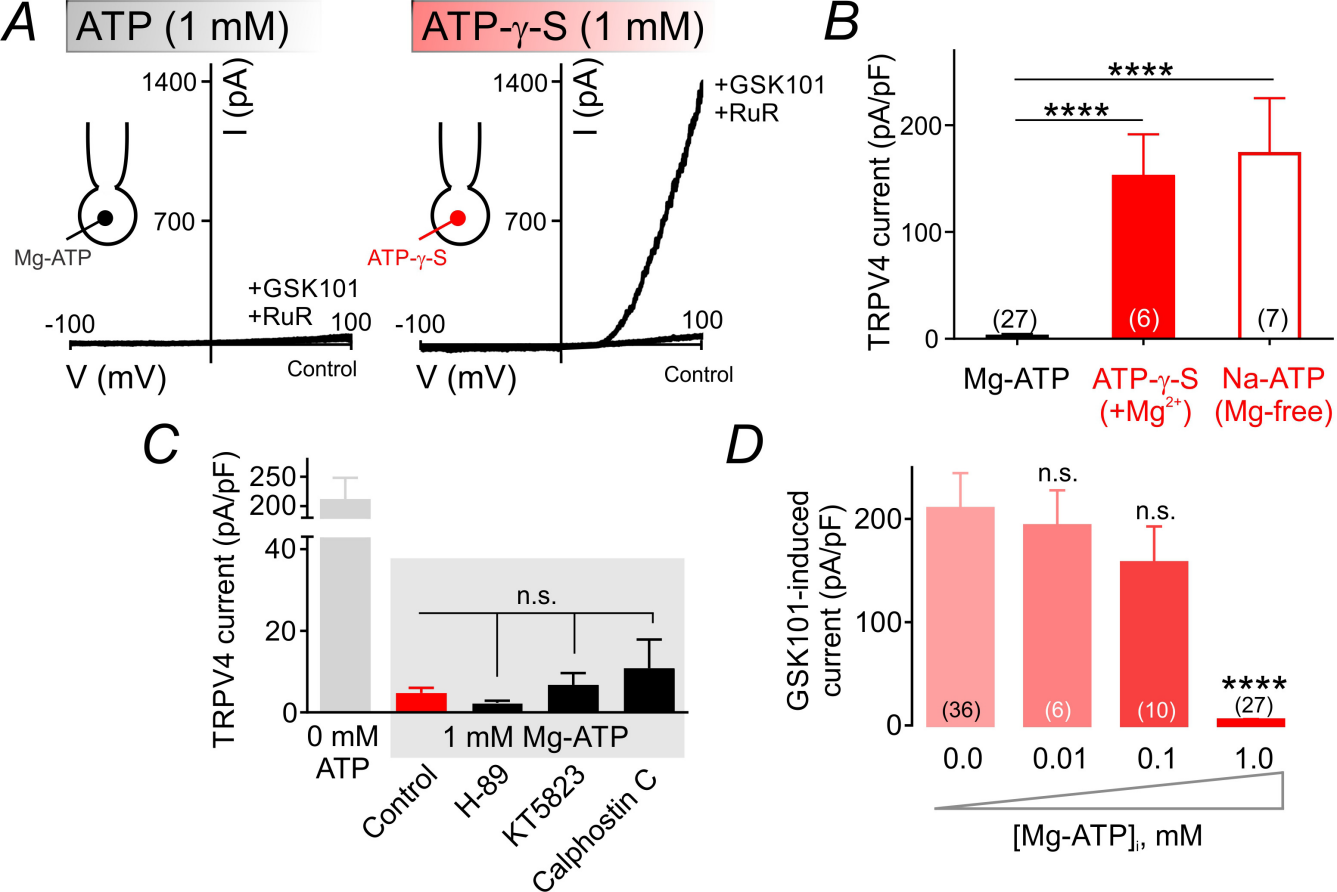
ATP hydrolysis is required for ATP-mediated suppression of TRPV4 channel activity. (**A**) Representative traces of current-voltage relationships in cECs recorded using voltage ramps (−100 to 100 mV) before and after the application of 100 nM GSK101 and 1 µM RuR. cECs were dialyzed with 1 mM Mg-ATP (*left*) or 1 mM Mg-ATP-γ-S (*right*). (**B**) Summary data showing GSK101 (100 nM)-induced outward currents at 100 mV in cECs dialyzed with Mg-ATP (1 mM), Mg-ATP-γ-S (1 mM) or Na-ATP (1 mM, in Mg^2+^ free solution). A minimum duration of 5 min after the application of GSK101 was allowed for outward TRPV4 current to develop in each cEC. Data are presented as means ± SEM (****p<0.0001 vs. Mg-ATP, one-way ANOVA followed by Dunnett’s multiple comparisons test). (**C**) TRPV4 outward currents induced by 100 nM GSK101 at 100 mV, recorded from dialyzed cECs (0 and 1 mM Mg-ATP). Mg-ATP–dialyzed cECs (gray shadow) were pre-treated with inhibitors of PKA (H-89, 1 µM), PKG (KT5823, 1 µM) or PKC (calphostin C, 0.5 µM) for ~10–15 min prior to GSK101 application, or left untreated (control). Data are presented as means ± SEM (n.s. denotes not significant vs. control Mg-ATP, one-way ANOVA, Dunnett’s multiple comparisons test, n = 6–24). (**D**) Summary data showing the effect of raising intracellular Mg-ATP concentration on GSK101-induced TRPV4 currents. Data are means ± SEM (****p<0.0001, one-way ANOVA followed by Dunnett’s multiple comparisons test). 10.7554/eLife.38689.011Figure 2—source data 1.Numerical data that were used to generate the chart in Figure 2B. 10.7554/eLife.38689.012Figure 2—source data 2.Numerical data that were used to generate the chart in Figure 2C. 10.7554/eLife.38689.013Figure 2—source data 3.Numerical data that were used to generate the chart in Figure 2D.

The Mg^2+^ salt of ATP is readily hydrolyzable and can be utilized by kinases (O'Rourke, 1993). To test whether TRPV4 suppression requires ATP hydrolysis, we replaced ATP in the patch pipette with the poorly hydrolyzable analog, ATP-γ-S (1 mM). Under these conditions, GSK101 (100 nM) increased TRPV4 currents to same extent as observed in cells dialyzed with 0 mM Mg-ATP, indicating that ATP hydrolysis is required for channel inhibition (Figure 2A,B; Figure 2—source data 1). This implies an energy-consuming process, and suggests the involvement of an enzyme, possibly a kinase. Coordination of Mg^2+^ is critical for kinase-mediated phosphoryl transfer reactions (O'Rourke, 1993; Ryves and Harwood, 2001; Zakharian et al., 2011). Thus, the prediction is that ATP would be unable to inhibit TPRV4 currents in the absence of the cofactor Mg^2+^ if a kinase underlies Mg-ATP–mediated TRPV4 inhibition. Consistent with this prediction, replacing Mg-ATP in the patch pipette with the Na^+^ salt of ATP (Na-ATP, 1 mM) while eliminating Mg^2+^ from the pipette solution abolished the suppressive effect of ATP (Figure 2B; Figure 2—source data 1), suggesting the contribution of a kinase to this process.

On the basis of these observations, we next tested the involvement of protein kinases by bath-applying the protein kinase A (PKA) inhibitor H-89 (1 µM), protein kinase G (PKG) inhibitor KT5823 (1 µM), or protein kinase C (PKC) inhibitor calphostin C (0.5 µM). We then monitored outward TRPV4-mediated currents as described above. None of these inhibitors affected currents in cells dialyzed with Mg-ATP (Figure 2C; Figure 2—source data 2), ruling out a role for these protein kinases in TRPV4 inhibition. Further support for this conclusion is provided by the observation that lower concentrations of Mg-ATP (10 and 100 µM)—which activate the majority of protein kinases (Knight and Shokat, 2005)—failed to effectively inhibit TRPV4 currents in cECs (Figure 2D; Figure 2—source data 3).

### ATP-dependent suppression of TRPV4 channels is mediated by PIP_2_

**Figure 3.**
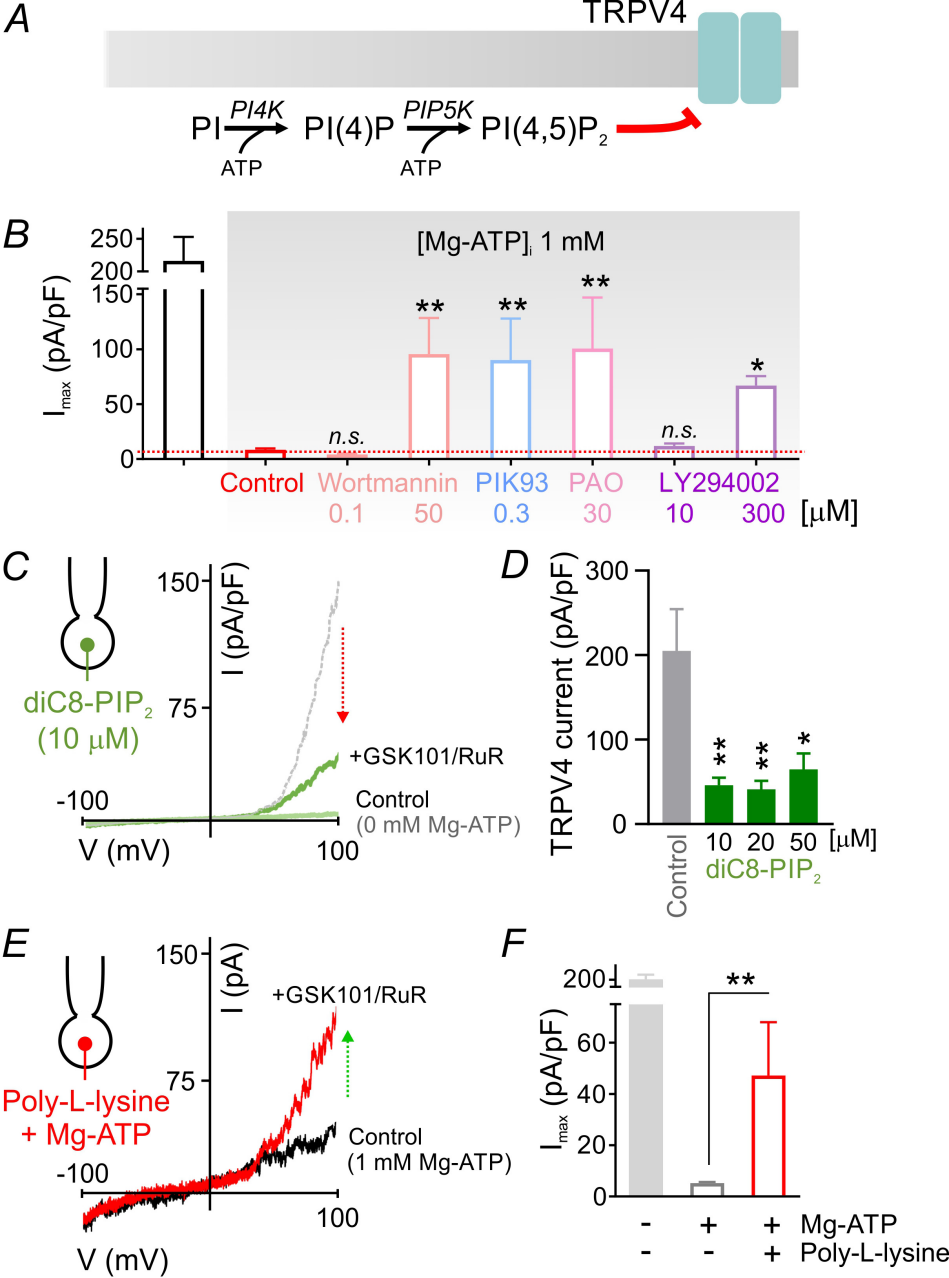
PIP_2_ mediates tonic inhibition of capillary TRPV4 channels. (**A**) Schematic diagram showing the ATP-dependent synthesis steps leading to the production of PIP_2_. (**B**) Average maximum outward TRPV4 current induced by 100 nM GSK101, recorded in cECs at 100 mV using the conventional whole-cell configuration. cECs dialyzed with 1 mM Mg-ATP were treated for ~10 min with wortmannin (0.1, 50 µM), PIK93 (0.3 µM), PAO (30 µM) or LY294002 (10, 300 µM), or were left untreated (control). A minimum duration of 10–15 min after the application of GSK101 was allowed for outward TRPV4 current to develop in each cEC. Data are means ± SEM (**p<0.01, *p<0.05 vs. control Mg-ATP, one-way ANOVA followed by Dunnett’s multiple comparisons test; n = 6–27). (**C**) Traces of current-voltage relationship obtained from a cEC dialyzed with 10 µM diC8-PIP_2_ and 0 mM Mg-ATP using a voltage ramp (−100 to 100 mV) before and after (green) the application of GSK101 (100 nM) and RuR (1 µM). The dotted gray trace is a representative GSK101-induced current recorded from a control cEC dialyzed with 0 µM diC8-PIP_2_ and 0 mM Mg-ATP. (**D**) Summary data showing GSK101 (100 nM)-induced currents at 100 mV in cECs dialyzed with different concentrations of diC8-PIP_2_ (10, 20, 50 µM) or 0 µM phosphoinositide (control). The pipette solution lacked Mg-ATP in all groups. GSK101-evoked outward currents developed over ~5 min. Data are presented as means ± SEM (*p<0.05, **P<0.01, one-way ANOVA followed by Dunnett’s multiple comparisons test; n = 10–18). (**E, F**) Representative trace (**E**) and summary data showing GSK101-induced currents in cECs dialyzed with 1 mM Mg-ATP and poly-L-lysine (3 µg/ml). A duration of 10 min was allowed after the application of GSK101 for outward TRPV4 current to develop in each cEC. Data in F are presented as means ± SEM (**p<0.01, unpaired Student’s t-test; n = 8–18). 10.7554/eLife.38689.017Figure 3—source data 1.Numerical data that were used to generate the chart in Figure 3B. 10.7554/eLife.38689.018Figure 3—source data 2.Numerical data that were used to generate the chart in Figure 3D. 10.7554/eLife.38689.019Figure 3—source data 3.Numerical data that were used to generate the chart in Figure 3F.

Lipid kinases, like protein kinases, also hydrolyze ATP, but typically require higher ATP concentrations (Hilgemann, 1997; Knight and Shokat, 2005; Xie et al., 1999). The requirement for millimolar Mg-ATP concentrations to effectively inhibit TRPV4 currents (Figure 1C, Figure 2D) is thus more consistent with lower ATP-affinity lipid kinases like those involved in phosphoinositide synthesis (Balla and Balla, 2006). In this cascade (Figure 3A), phosphatidylinositol 4-kinase (PI4K) utilizes ATP to phosphorylate phosphatidylinositol (PI), converting it to phosphatidylinositol 4-phosphate (PIP), which in turn is phosphorylated to PIP_2_ by phosphatidylinositol 4-phosphate 5-kinase (PIP5K), a step that also requires ATP. Thus, the cellular levels of PIP_2_ are maintained through an ATP-hydrolysis–dependent process (Baukrowitz et al., 1998; Hilgemann, 1997; Suh and Hille, 2002). To assess the possible involvement of this pathway in capillary TRPV4 suppression, we tested the effects of four structurally unrelated pharmacological inhibitors of PI4K: wortmannin (50 µM), PIK93 (300 nM), phenylarsine oxide (PAO; 30 µM), and LY294002 (300 µM). In each case, inhibitors of PIP_2_ synthesis significantly reversed TRPV4 channel inhibition by ATP (Figure 3B; Figure 3—source data 1), despite the high intracellular Mg-ATP concentration (1 mM). Notably, at concentrations below those necessary to inhibit PI4K (but sufficient to suppress phosphoinositide 3-kinases), the inhibitors wortmannin (0.1 µM) and LY294002 (10 µM) failed to increase TRPV4 current in cECs (Figure 3B; Figure 3—source data 1). Collectively, these observations indicate the involvement of the ATP/PI4K regulatory axis, and presumably PIP_2_, in suppressing TRPV4 channels in cECs.

**Figure 3—figure supplement 1.**
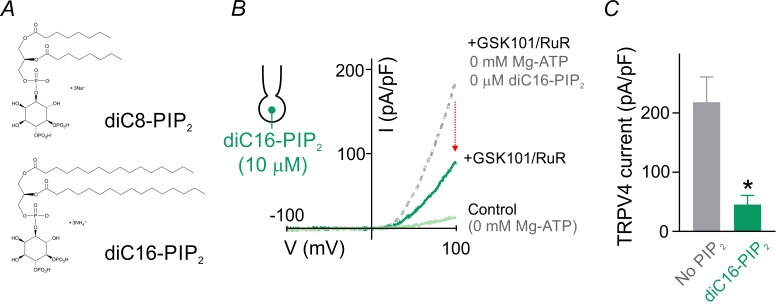
The long-acyl chain PIP_2_, diC16-PIP_2_, suppresses TRPV4 currents. (**A**) Chemical structures of short (dioctanoyl, diC8-PIP_2_) and long (dipalmitoyl, diC16-PIP_2_) acyl chain PIP_2_ salts used in this study. (**B**) Current-voltage relationship represents currents obtained from a cEC dialyzed with 10 µM diC16-PIP_2_ and 0 mM Mg-ATP before (light green) and after (dark green) the application of GSK101 (100 nM) and RuR (1 µM), recorded using a voltage ramp (−100 to 100 mV). Outward current developed over 5 min. The dotted gray trace is a representative GSK101-induced current recorded from a control cECs dialyzed with 0 mM diC16-PIP_2_ and 0 mM Mg-ATP. (**C**) Summary data showing GSK101 (100 nM)-induced currents at 100 mV in cECs dialyzed with diC16-PIP_2_ (10 µM) or 0 µM phosphoinositide (control). The pipette solution lacked Mg-ATP in both groups. Data are presented as means ± SEM (*p<0.05, unpaired Student’s t-test; n = 3–4 cECs).

**Figure 3—figure supplement 2.**
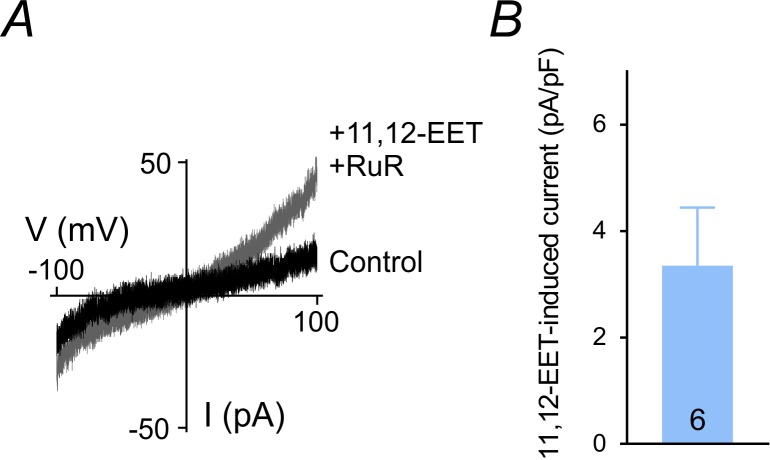
11,12-EET-induced currents in cECs. (**A**) Current-voltage relationship represent currents obtained from a cEC dialyzed with 0 µM diC8-PIP_2_ and 0 mM Mg-ATP before (control) and after the application of 11,12-EET (1 µM) and RuR (1 µM), recorded using 300 ms voltage ramps (−100 to 100 mV). (**B**) Summary data showing 11,12-EET-induced outward currents at 100 mV in dialyzed cECs based on experiments in *A* (n = 6 cECs).

PIP_2_ is a well-established regulator of membrane proteins, including ion channels (Hille et al., 2015; Huang et al., 1998; Nilius et al., 2008). To directly test whether PIP_2_ inhibits TRPV4 channels in cECs, we introduced the water-soluble, short acyl chain dioctanoyl PIP_2_ (diC8-PIP_2_) via the patch pipette. In the absence of Mg-ATP, diC8-PIP_2_ inhibited GSK101-induced TRPV4 currents, reducing them by 70–80% at concentrations of 10 to 50 µM (Figure 3C,D; Figure 3—source data 2). Similar inhibition was observed with the longer-chain PIP_2_ analog, diC16-PIP_2_ (10 µM) (Figure 3—figure supplement 1). Moreover, scavenging endogenous, negatively charged PIP_2_ with intracellular poly-L-lysine (3 µg/ml, molecular weight 15,000–30,000), included in the patch pipette, attenuated Mg-ATP effects on GSK101-induced TRPV4 currents (Figure 3E,F; Figure 3—source data 3). Taken together, these data show that PIP_2_ inhibits both GSK101-induced and constitutive TRPV4 channel activity in cECs.

### G_q_PCR activation relieves TRPV4 suppression in cECs by promoting PIP_2_ hydrolysis

**Figure 4.**
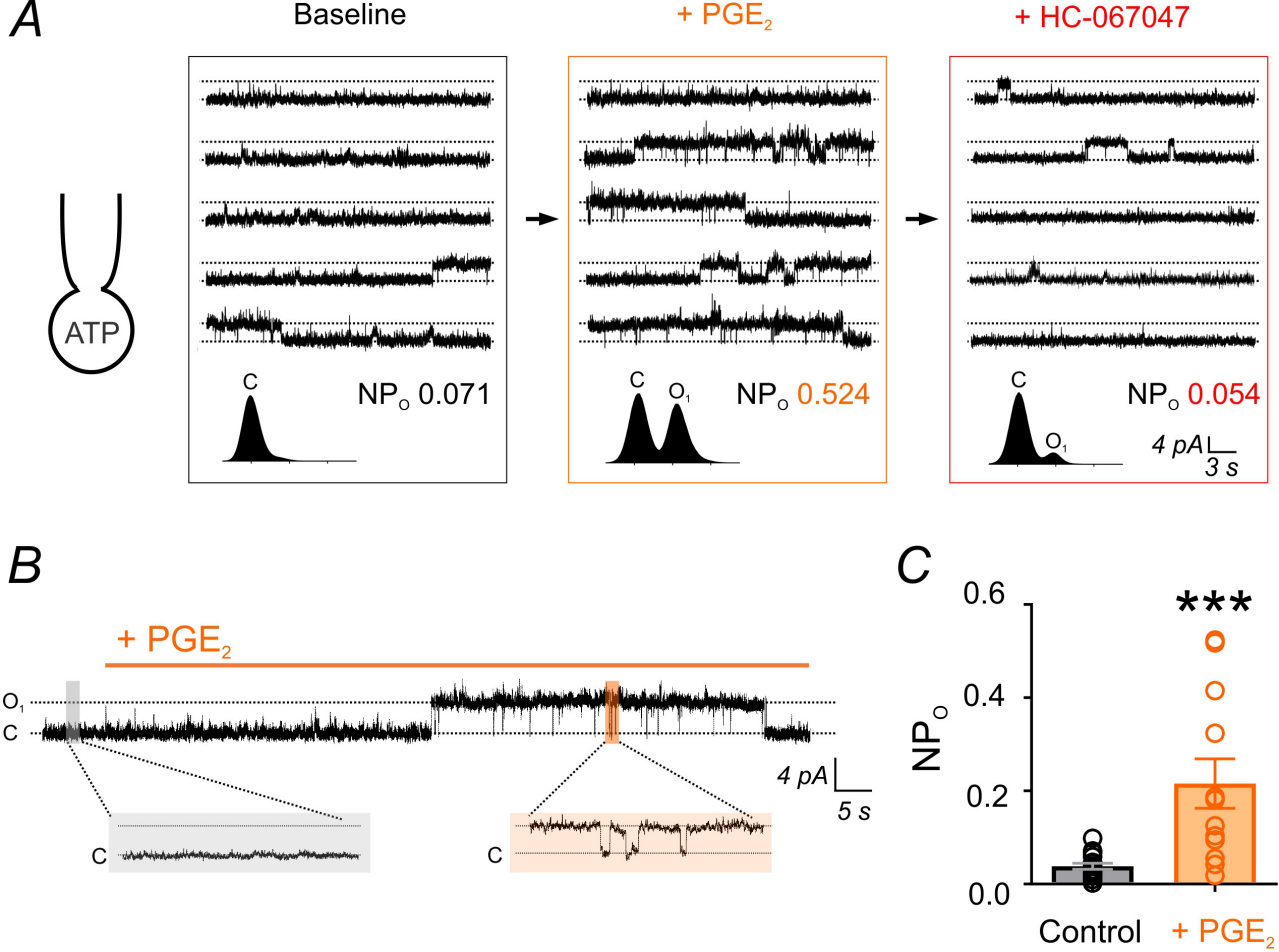
PGE_2_ enhances TRPV4 channel activity. (**A**) *Top:* Representative conventional whole-cell recordings from a cEC dialyzed with 1 mM Mg-ATP in the absence of GSK101 and held at a membrane potential of +50 mV. Quantal outward K^+^ currents (unitary current, 4.6 pA; sampling rate, 20 kHz; lowpass filter frequency, 1 kHz), reflecting single-channel openings, were recorded before (baseline) and after the consecutive application of PGE_2_ (2 µM) and HC-067047 (1 µM). *Bottom:* Corresponding amplitude histograms and open probability (NP_O_) values. (**B, C**) Representative trace (**B**) and individual-value plot (**C**) of TRPV4 NP_O_ in cECs (dialyzed with 1 mM Mg-ATP, held at +50 mV) in the absence (control; n = 16) and presence (n = 12) of 2 µM PGE_2_. Data in *C* are means (column bars) ± SEM (error bars, ***p<0.001, unpaired Student’s t-test). Each data point represents a recording from a cEC; the average duration of each recording was 5 min. 10.7554/eLife.38689.021Figure 4—source data 1.Numerical data that were used to generate the chart in Figure 4C.

The primary mechanism responsible for reducing PIP_2_ levels is activation of G_q_PCRs and subsequent PLC-mediated PIP_2_ hydrolysis. To determine whether endothelial G_q_PCR-activation–mediated PIP_2_ depletion relieves the inhibition of TRPV4 channels, we examined the effects of G_q_PCR agonists on constitutive single-channel TRPV4 currents in cECs under simulated physiological conditions (i.e. dialyzed with Mg-ATP) (Figure 1D). We first tested the effects of prostaglandin E2 (PGE_2_), a postulated NVC agent that acts on G_q_-coupled EP_1_ receptors in cECs to deplete PIP_2_ (Harraz et al., 2018) and has previously been proposed to act through G_s_-protein-coupled EP_2_ and EP_4_ receptors to cause vasodilation (Lacroix et al., 2015; Zonta et al., 2003). PGE_2_ significantly increased TRPV4 single-channel open probability in the absence of GSK101 (Figure 4A,B), increasing NP_O_ ~6 fold (Figure 4C; Figure 4—source data 1). This enhancement was similar to that observed under conditions in which intracellular Mg-ATP was excluded from the pipette solution (in the absence of either a receptor agonist or GSK101; Figure 1D), suggesting that PGE_2_ acts through PIP_2_ depletion to relieve PIP_2_-mediated inhibition and restore TRPV4 channel activity.

**Figure 5.**
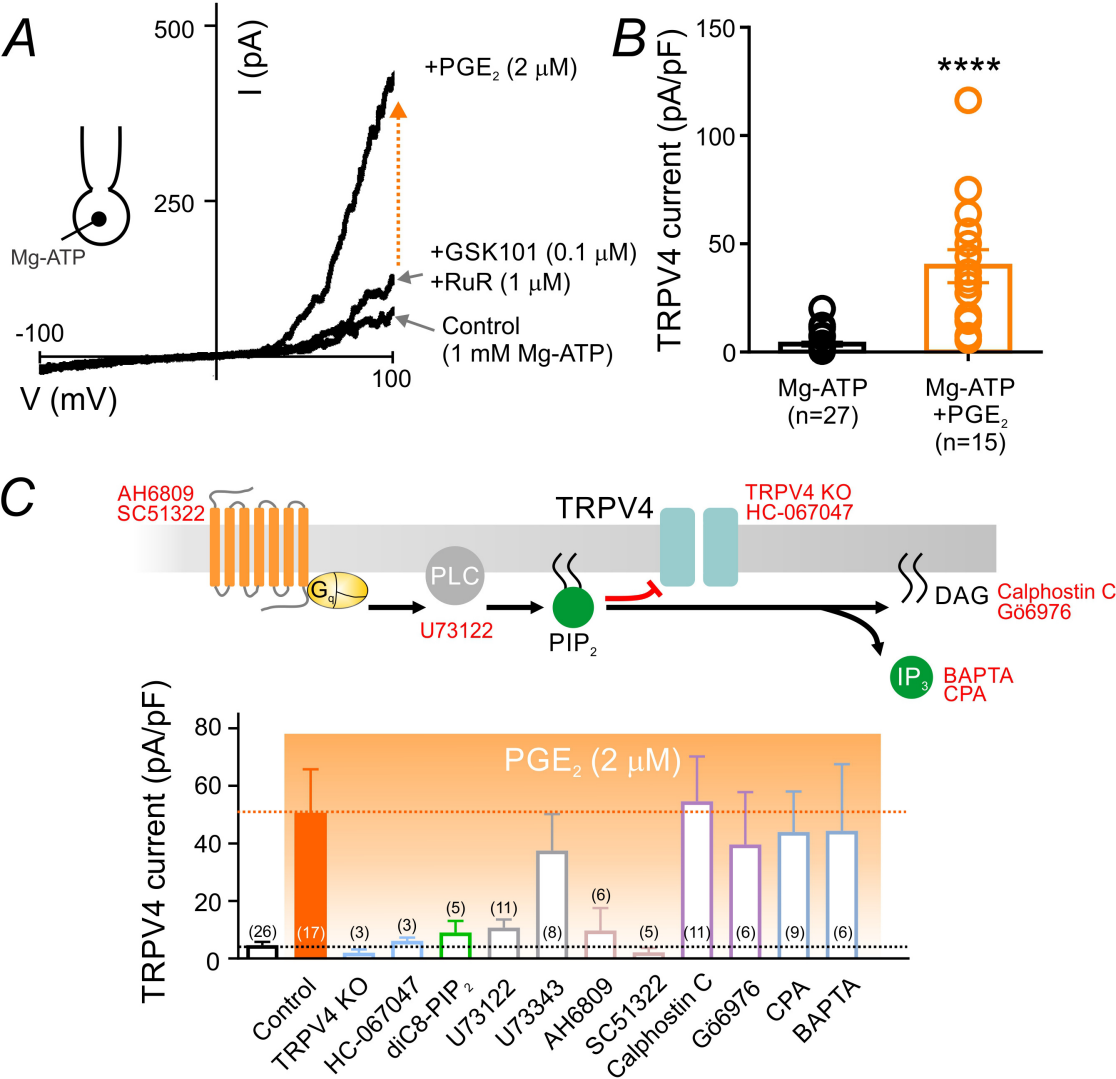
PGE_2_ relieves PIP_2_-mediated TRPV4 channel suppression. (**A**) Representative current-voltage plots obtained from a cEC dialyzed with 1 mM Mg-ATP and treated consecutively with GSK101 (100 nM) and RuR (1 µM) followed by 2 µM PGE_2_. (**B**) Summary individual-value plot of GSK101-induced TRPV4 currents at 100 mV in cECs dialyzed with 1 mM Mg-ATP in the absence (black; n = 27) and presence (orange; n = 15) of 2 µM PGE_2_. Incubation of cECs with PGE_2_ lasted ~15 min. Data in *B* are means (column bars) ± SEM (error bars, ****p<0.0001, unpaired Student’s t-test). (**C**) *Top:* Schematic diagram showing the G_q_PCR-dependent hydrolysis of PIP_2_ and the interventions used to test different components of the proposed pathway. *Bottom:* Summary data showing GSK101 (100 nM)-induced currents recorded at 100 mV in cECs dialyzed with 1 mM Mg-ATP. Currents were recorded in the absence and presence of 2 µM PGE_2_ (orange shading), with or without (control) the indicated interventions. Concentrations (and application method): HC-067047, 1 µM (bath); diC8-PIP_2_, 10 µM (pipette), U73122, 10 µM (bath); U73343, 10 µM (bath); AH6809, 10 µM (bath); SC51322, 1 µM (bath); calphostin C, 0.5 µM (bath); Gö6976, 1 µM (bath); CPA, 30 µM (bath); BAPTA, 5.4 mM (pipette). For bath application, pharmacological agents were added 10–15 min before PGE_2_ application. 10.7554/eLife.38689.025Figure 5—source data 1.Numerical data that were used to generate the chart in Figure 5B. 10.7554/eLife.38689.026Figure 5—source data 2.Numerical data that were used to generate the chart in Figure 5C.

To confirm that PIP_2_ depletion underlies PGE_2_-induced activation of TRPV4 channels, we next employed a series of conventional whole-cell recordings and pharmacological interventions to test the different components in the proposed pathway. In these experiments, cECs were dialyzed with 1 mM Mg-ATP—a maneuver sufficient to significantly suppress TRPV4 channels even in the presence of 100 nM GSK101 (Figure 1A,C). As predicted, application of PGE_2_ (2 µM) in the presence of GSK101 greatly increased TRPV4 currents in cECs dialyzed with Mg-ATP (Figure 5A,B; Figure 5—source data 1). Currents evoked by PGE_2_ application were inhibited by the TRPV4 antagonist HC-067047 (1 µM) and were absent in cECs from TRPV4^-/-^ animals, confirming that they are mediated by TRPV4 channels (Figure 5C; Figure 5—figure supplement 1). Introduction of the PIP_2_ analog diC8-PIP_2_ (10 µM) in the pipette solution or inhibition of PLC using U73122 (10 µM)—pharmacological interventions that serve to compensate for or prevent PLC-dependent PIP_2_ degradation, respectively—prevented the increase in TRPV4 currents by PGE_2_ (Figure 5C; Figure 5—figure supplement 1; Figure 5—source data 2). The non-selective prostanoid receptor (EP_1_/EP_2_/EP_3_) antagonist AH6809 (10 µM) also prevented this effect of PGE_2_, as did the selective EP_1_ antagonist SC51322 (1 µM), suggesting that PGE_2_ acts through the EP_1_ receptor. U73343, the inactive analog of the PLC inhibitor U73122, did not alter PGE_2_ effects (Figure 5C; Figure 5—source data 2). Collectively, these observations suggest that PGE_2_ signals through the EP_1_-PLC pathway to deplete PIP_2_ and thereby relieve PIP_2_-mediated TRPV4 inhibition.

**Figure 5—figure supplement 1.**
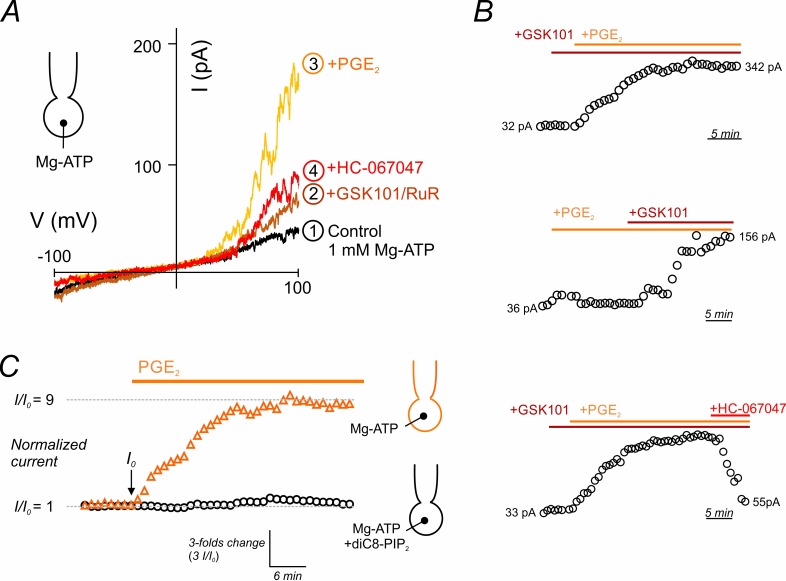
PGE_2_ relieves TRPV4 current inhibition. (**A**) Current-voltage relationship recorded in a cEC dialyzed with 1 mM Mg-ATP using voltage ramps (−100 to 100 mV). The circled numbers indicated the sequential and cumulative application of GSK101 (100 nM)+RuR (1 µM) followed by 2 µM PGE_2_ and lastly, the TRPV4 blocker HC-067047 (1 µM). (**B**) Representative scatter plots of peak current amplitudes (at 100 mV) in three cECs dialyzed with 1 mM Mg-ATP and treated with 100 nM GSK101 followed by 2 µM PGE_2_ (or PGE_2_ first followed by GSK101). Note that PGE_2_-induced activation of TRPV4 current plateaued after ~13–15 min. (**C**) Scatter plots of normalized GSK101-induced outward currents (at 100 mV), normalized to the current at zero time, *I*_0_ (when 2 µM PGE_2_ was applied), in two cECs, one dialyzed with 1 mM Mg-ATP (orange symbols) and the other dialyzed with 1 mM Mg-ATP +10 µM diC8-PIP_2_ (black symbols). Currents normalized to *I*_0_ are presented before and after the application of PGE_2_ for 35 min.

**Figure 5—figure supplement 2.**
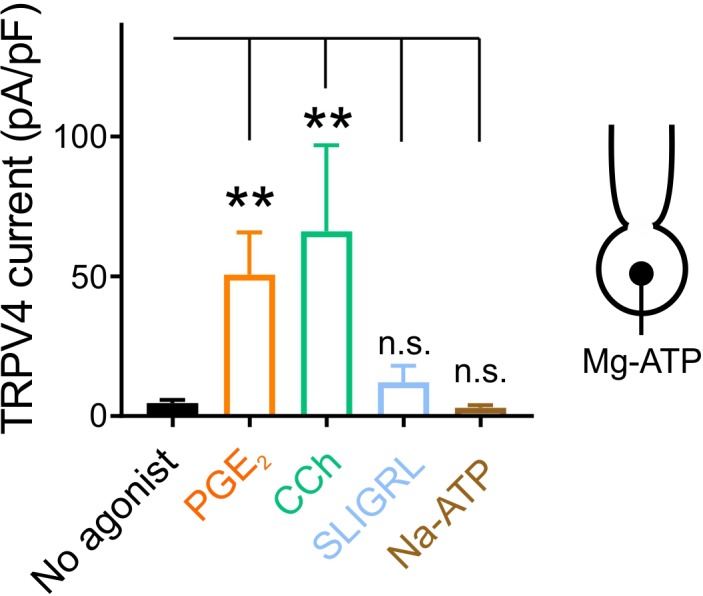
Different G_q_PCR agonists differentially regulate TRPV4 activity. Summary data showing TRPV4 currents evoked by 100 nM GSK101 at 100 mV in Mg-ATP (1 mM)-dialyzed cECs. Cells were treated with GSK101 alone (no agonist) or together with the G_q_PCR agonist PGE_2_ (2 µM), carbachol (CCh; 10 µM), SLIGRL (5 µM), or Na-ATP (50 µM). cECs were incubated with different agonists for 15 min while TRPV4 current development was monitored. Data are presented as means ± SEM (**p<0.01, one-way ANOVA followed by Dunnett’s multiple comparisons test; n = 11–17 cECs).

The PIP_2_ breakdown products, DAG and IP_3_, stimulate PKC activity and promote Ca^2+^ release from the endoplasmic reticulum, respectively. To rule out the involvement of these pathways in the PGE_2_-induced increase in TRPV4 currents, we first tested the effects of the PKC inhibitors, Gö6976 (1 µM) and calphostin C (0.5 µM), and found that neither altered PGE_2_-induced activation of TRPV4 channels (Figure 5C; Figure 5—source data 2). To determine whether intracellular Ca^2+^ signals downstream of EP_1_-PLC activation by PGE_2_ are involved in TRPV4 disinhibition, we blocked Ca^2+^ reuptake into intracellular stores by inhibiting the sarcoplasmic/endoplasmic reticulum Ca^2+^ ATPase (SERCA) pump with cyclopiazonic acid (CPA; 30 µM) or by rapidly chelating cytoplasmic Ca^2+^ with BAPTA (5.4 mM). Neither maneuver attenuated PGE_2_-induced disinhibition of TRPV4 channel activity (Figure 5C; Figure 5—source data 2), arguing against a major contribution of DAG-PKC or IP_3_-Ca^2+^ signaling to this effect. Taken together, our data show that PGE_2_ activates EP_1_ receptors and downstream PLC to deplete PIP_2_ and thereby relieve TRPV4 channel inhibition, independently of PIP_2_ metabolites.

Carbachol (10 µM), which can signal through G_q_-coupled muscarinic receptors, induced an increase in TRPV4 currents comparable to that produced by PGE_2_ (Figure 5—figure supplement 2), indicating that G_q_PCR-mediated TRPV4 activation is not restricted to prostanoid EP_1_ receptors. However, neither the purinergic receptor agonist, ATP (50 µM), nor the protease-activated receptor-2 (PAR2)-activating peptide, SLIGRL-NH_2_ (5 µM), affected TRPV4 channel activity (Figure 5—figure supplement 2). The reason for these apparent differential effects of various G_q_PCRs is currently unclear, but could reflect differences in expression levels or cellular localization of the corresponding receptors, or receptor desensitization owing to ligand-dependent proteolysis (PAR2) or receptor internalization (e.g. P2Y subtypes)(Cho et al., 2005a, 2005b; Dickson et al., 2013; Jung et al., 2016; Sromek and Harden, 1998; Vanlandewijck et al., 2018).

### G_q_PCR signaling as a ‘switch’ that shifts the balance between TRPV4 and Kir2.1 signaling

**Figure 6.**
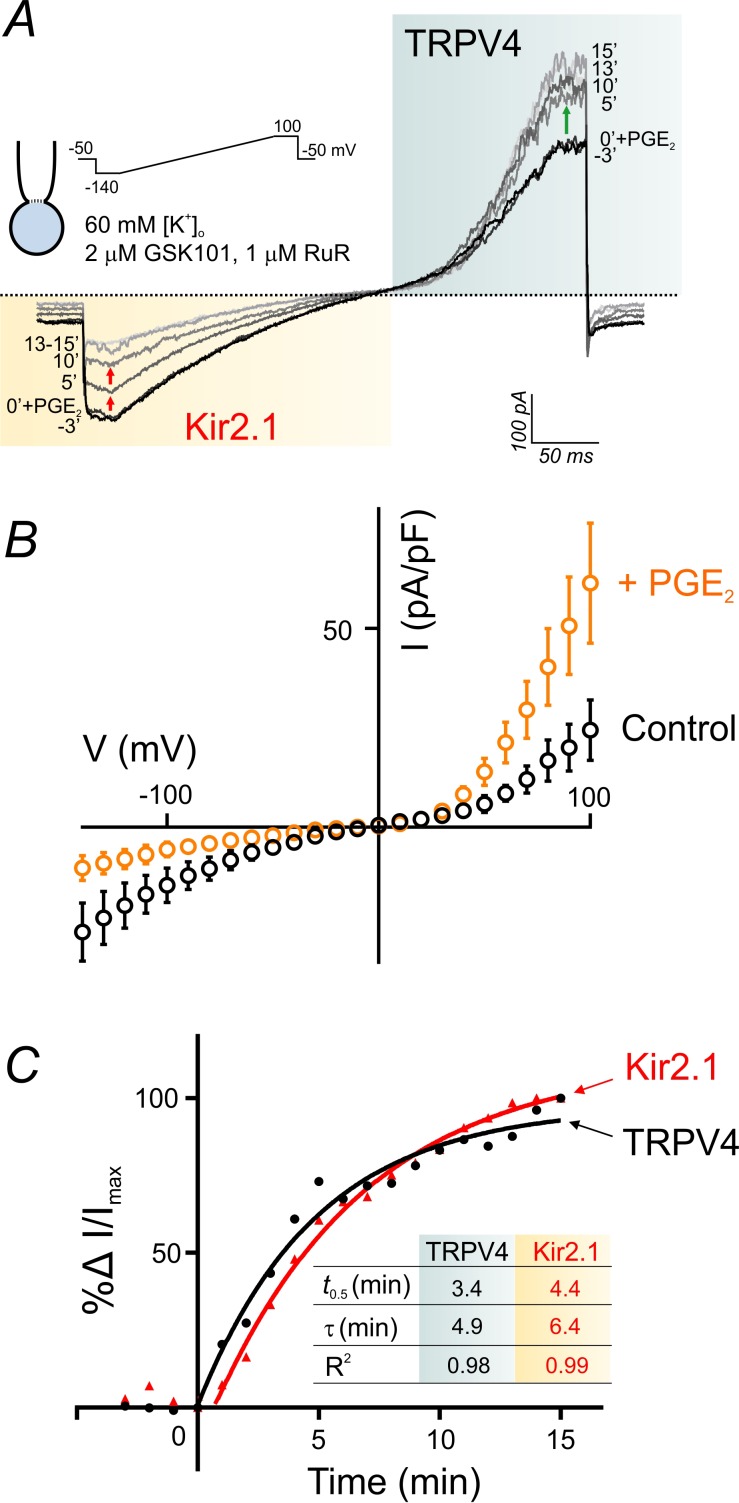
PGE_2_ simultaneously and reciprocally regulates TRPV4 and Kir2.1 channel activities. (**A**) Representative traces illustrate simultaneous recordings of Kir2.1 (inward) and TRPV4 (outward) currents in a cEC obtained using the perforated whole-cell configuration. Voltage ramps (300 ms, −140 to 100 mV) were used and the cEC was bathed in a 60 mM [K^+^]_o_ solution supplemented with 2 µM GSK101 and 1 µM RuR. Traces represent currents before and for a duration of 15 min after the application of 2 µM PGE_2_. (**B**) Averaged current-voltage relationship (n = 5 cECs) corresponding to the experiment in *A*, before (control) and after (maximum changes at 15 min) application of PGE_2_. (**C**) Summary data showing the kinetics of TRPV4 current enhancement (black) and Kir2.1 current decline (red) following application of 2 µM PGE_2_ onto cECs (as in *A*) at room temperature. Points are average percentage change in normalized currents before and over 15 min after PGE_2_ application (n = 5). Curves are best fits of exponential change (rise: TRPV4; decay: Kir2.1). *Inset table:* kinetic parameters based on the two curve fits. Changes in currents plateaued ~13 min after the application of PGE_2_. 10.7554/eLife.38689.031Figure 6—source data 1.Numerical data that were used to generate the chart in Figure 6B. 10.7554/eLife.38689.032Figure 6—source data 2.Numerical data that were used to generate the chart in Figure 6C.

**Figure 7.**
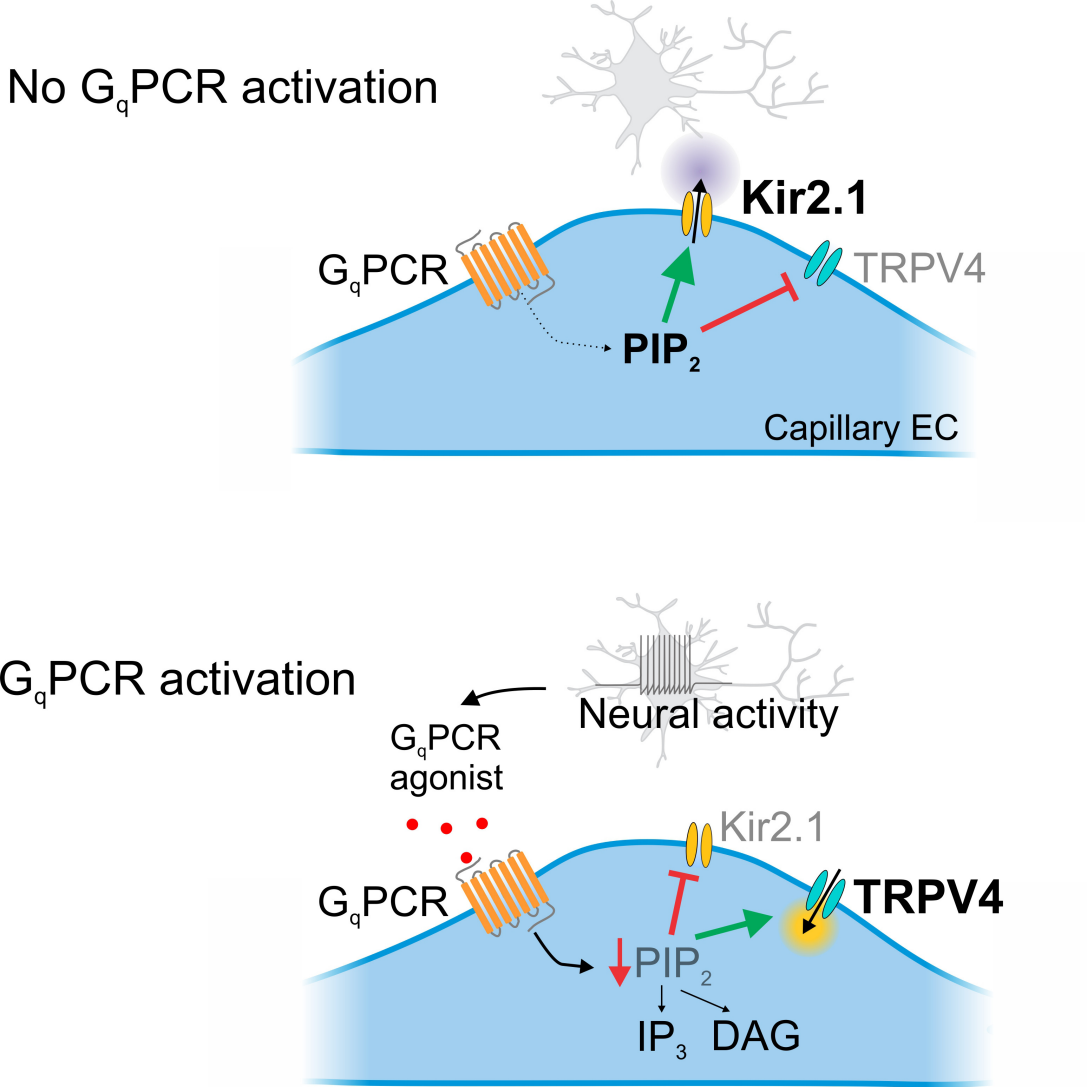
Cartoon representation of G_q_PCR-mediated reciprocal effects on capillary ion channel activity. Schematic diagram summarizing the proposed mechanism. *Top*: In the absence of G_q_PCR stimulation, endogenous PIP_2_ levels are sufficient to tonically inhibit TRPV4 channels and maintain Kir2.1 channel activity. *Bottom*: G_q_PCR activation with an agonist stimulates PIP_2_ hydrolysis, resulting in the loss of PIP_2_-mediated maintenance of Kir2.1 activity and inhibition of TRPV4 activity.

PIP_2_ levels are key to the maintenance and activation of inward rectifier K^+^ channels, as reported recently by our group (Harraz et al., 2018) and others (D'Avanzo et al., 2010; Hansen et al., 2011; Huang et al., 1998). Our current (Figure 5) and previous (Harraz et al., 2018) findings indicate that G_q_PCR-activation–induced depletion of PIP_2_ exerts opposite effects on endothelial TRPV4 (*activation*) and Kir2.1 (*inhibition*) channels. In fact, G_q_PCR activation in cECs deactivates Kir2.1 currents through PIP_2_ hydrolysis and cripples capillary-to-arteriolar electrical signaling (Harraz et al., 2018). It is thus conceivable that G_q_PCR-mediated signaling could shift the balance between TRPV4 and Kir2.1 signaling in brain capillaries through perturbation of endothelial PIP_2_ levels. To test this, we designed an experiment that allowed simultaneous monitoring of TRPV4 and Kir2.1 currents in the context of G_q_PCR-mediated changes in endothelial PIP_2_ levels. Using the perforated-patch configuration to maintain physiological levels of ATP and PIP_2_, and bathing cECs in a solution containing an extracellular K^+^ concentration ([K^+^]_o_) of 60 mM to facilitate monitoring of Kir2.1 currents, we evoked TRPV4 currents with 2 µM GSK101, a concentration sufficient to sub-maximally activate an outward current in this configuration (in the presence of 1 µM RuR to block inward TRPV4 current). In this setting, application of PGE_2_ (2 µM) enhanced TRPV4 currents and simultaneously inhibited Kir2.1 currents (Figure 6A,B; Figure 6—source data 1) compared with matching time controls, which showed no significant change in either current (Figure 6—figure supplement 1). The onset of changes in TRPV4 and Kir2.1 currents in response to PGE_2_ was rapid (<60 s). A kinetic analysis revealed that TRPV4 activation and Kir2.1 suppression were kinetically comparable, with times for half-maximal change in activity (*t*_0.5_) of 3.4 min for TRPV4 and 4.4 min for Kir2.1 channels, and corresponding time constants (τ) of 4.9 and 6.4 min, respectively (Figure 6C; Figure 6—source data 2). The muscarinic receptor agonist carbachol (10 µM) similarly facilitated TRPV4 currents and inhibited Kir2.1 currents (Figure 6—figure supplement 2). In contrast, purinergic receptor stimulation with ATP did not affect TRPV4 currents (Figure 5—figure supplement 2; Figure 6—figure supplement 3) despite inhibiting Kir2.1 currents (Figure 6—figure supplement 3), as we reported previously (Harraz et al., 2018). These findings collectively establish a potential mechanism by which PGE_2_ or other suitable G_q_PCR agonists, could alter the balance between electrical (hyperpolarizing) signaling mediated by Kir2.1 channels and TRPV4 channel signaling in capillaries through modulation of PIP_2_ levels (Figure 7).

**Figure 6—figure supplement 1.**
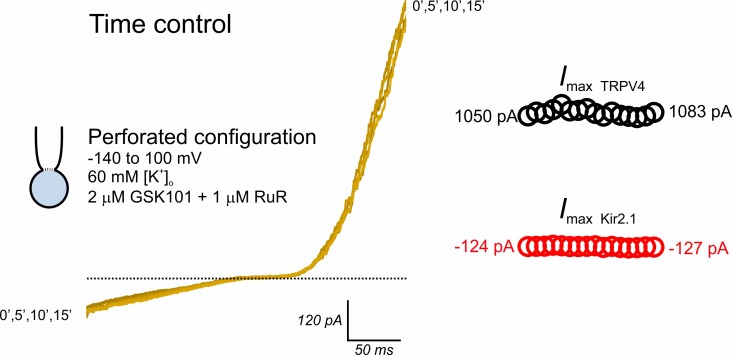
TRPV4 and Kir2.1 currents are preserved in cytoplasm-intact cECs. Representative traces obtained from a cEC using the same experimental protocol used in Figure 6 (perforated whole-cell configuration, 60 mM [K^+^]_o_, bath application of 2 µM GSK101 +1 µM RuR). Inward current reflects Kir2.1 activity, and outward current reflects TRPV4 activity. Traces (*left*) and scatter plots (*right*) track inward and outward currents over a duration of 15 min.

**Figure 6—figure supplement 2.**
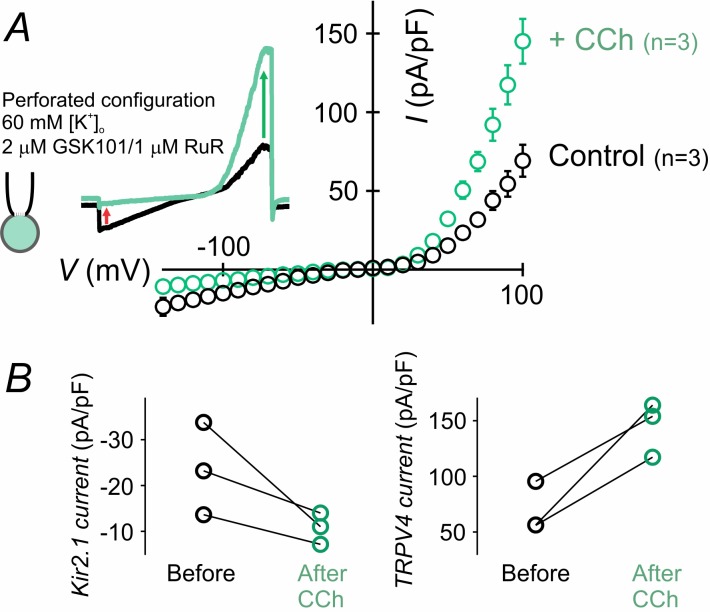
The muscarinic receptor agonist carbachol activates TRPV4 currents and inhibits Kir2.1 currents. (**A**) Representative traces and averaged current-voltage relationship for Kir2.1 (inward) and TRPV4 (outward) currents, simultaneously recorded in cECs using the perforated whole-cell configuration. The cEC was bathed in 60 mM [K^+^]_o_ solution supplemented with 2 µM GSK101 and 1 µM RuR, and currents were recorded using voltage ramps (300 ms, −140 to 100 mV). Traces and curves represent current values before and 15 min after the application of carbachol (CCh; 10 µM). (**B**) Paired scatter plots show maximum Kir2.1 (inward at −140 mV) and TRPV4 (outward at 100 mV) current densities before and 15 min after CCh (n = 3 each).

**Figure 6—figure supplement 3.**
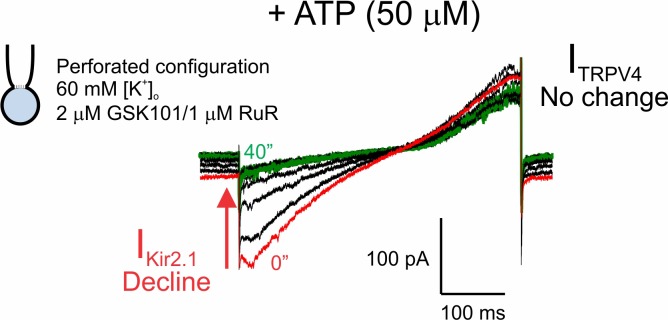
Purinergic receptor activation affects Kir2.1 channels but not TRPV4 channels. Representative traces of Kir2.1 (inward) and TRPV4 (outward) currents in a cEC, simultaneously recorded using the perforated whole-cell configuration. The cEC was bathed in 60 mM [K^+^]_o_ solution supplemented with 2 µM GSK101 and 1 µM RuR, and currents were recorded using voltage ramps (300 ms, −140 to 100 mV). Traces represent current values before and at various times up to 40 min after the bath-application of 50 µM Na-ATP.

## Discussion

We previously demonstrated that increases in [K^+^]_o_ associated with neuronal activity alter cerebral blood flow at the capillary level, showing that extracellular K^+^ activates capillary Kir2.1 channels, triggering a retrograde electrical (hyperpolarizing) signal that propagates upstream to dilate feeding arterioles and enhance blood flow to the active region (Longden et al., 2017). We have also shown that G_q_PCR-mediated PIP_2_ depletion inhibits capillary electrical signaling (Harraz et al., 2018), identifying a point of intersection between electrical signaling and endothelial G_q_PCR activity. In the present study, we show that cECs express TRPV4 channels, and demonstrate that these channels are tonically suppressed by basal levels of PIP_2_. Furthermore, we show that G_q_PCR activation relieves TRPV4 channel inhibition through PIP_2_ depletion. This paradigm introduces the phosphoinositide PIP_2_ as a master regulator of TRPV4 and Kir2.1 signaling in the capillary endothelium and highlights the ability of G_q_PCR activity to tune the balance of these signaling modalities to favor TRPV4 signaling (Figure 7).

The repertoire of functional ion channels in brain cECs remains incompletely characterized, although certain molecular features have recently come into focus. For instance, our evidence suggests that the inward rectifier Kir2.1 channel is the major K^+^ channel type in brain cECs (Longden et al., 2017), whereas Ca^2+^-sensitive SK and IK channels, which are present in arterial and arteriolar ECs and play a prominent role in regulating vascular tone (Ledoux et al., 2008; Sonkusare et al., 2012; Taylor et al., 2003), are not expressed in brain cECs (Longden et al., 2017). Notably, the identity of depolarizing (Na^+^ and/or Ca^2+^-permeable) channels in cECs, which we predict must be present to allow the membrane potential to reset to support repeated operation of our previously reported Kir2.1-dependent electrical signaling-based NVC mechanism, is not known. Our demonstration that the highly selective TRPV4 agonist, GSK101, induced currents in brain cECs that were eliminated by the TRPV4-specific antagonist, HC-067047, and were absent in cECs from TRPV4^-/-^ mice firmly establishes the presence of this non-selective cation channel in capillaries. In arterial and arteriolar ECs, TRPV4 channel-mediated Ca^2+^ influx is closely linked to activation of Ca^2+^-sensitive SK and IK channels and subsequent membrane potential hyperpolarization (Sonkusare et al., 2012). However, because cECs lack functional Ca^2+^-activated K^+^ channels (Longden et al., 2017), TRPV4-mediated influx of Na^+^/Ca^2+^ in these cells instead would lead to membrane potential depolarization (Behringer et al., 2017; Earley and Brayden, 2015). Collectively, these observations suggest that the TRPV4 channel is a major depolarizing element in cECs, although we cannot rule out the possibility that other, as yet unidentified, cation channels may contribute to membrane depolarization, as suggested by earlier studies (Csanády and Adam-Vizi, 2004; Popp and Gögelein, 1992; Csanády and Adam-Vizi, 2003). Our observations also highlight the fact that the functional role of TRPV4 channels is critically dependent on the expression and function of key associated proteins.

Intriguingly, and in striking contrast to the case in peripheral arterial ECs (Sonkusare et al., 2014; Sonkusare et al., 2012), the open probability of capillary TRPV4 channels in cECs was remarkably low under basal conditions and was increased by dialyzing out intracellular contents—the first link in the chain leading to our discovery that TRPV4 channels in cECs are intrinsically inhibited by intracellular ATP. It is well established that lipid kinases involved in PIP_2_ synthesis require millimolar ATP for their activity (Balla and Balla, 2006; Hilgemann, 1997; Knight and Shokat, 2005; Suer et al., 2001), and it has been amply demonstrated that PIP_2_ maintenance is dependent on ATP in multiple cell types (Suh and Hille, 2008; Ye et al., 2018; Zakharian et al., 2011), including cECs (Harraz et al., 2018). Three major lines of evidence presented here support the conclusion that cytosolic ATP-dependent maintenance of sustained, basal levels of PIP_2_ suppresses TRPV4 channel activity. First, millimolar concentrations of hydrolyzable ATP suppressed capillary TRPV4 channel activity. Second, inclusion of PIP_2_ analogs in the patch pipette inhibited TRPV4 currents. Third, scavenging PIP_2_ or inhibiting its synthesis abrogated ATP-mediated inhibition. Notably, PIP_2_ has been reported to directly interact with different residues on the TRPV4 N-terminus in heterologous expression systems (Garcia-Elias et al., 2013; Takahashi et al., 2014). However, these studies reached discrepant conclusions, with one suggesting that direct binding of PIP_2_ to the ankyrin repeat domain of the TRPV4 channel inhibits different modes of TRPV4 activation (Takahashi et al., 2014) and the other reporting that PIP_2_ is necessary for heat-, hypotonicity- or epoxyeicosatrienoic acid (EET)-induced channel activation by facilitating structural rearrangements of the channel (Garcia-Elias et al., 2013). These discrepancies may reflect divergent effects of PIP_2_ on channel behavior through binding to multiple sites in the channel. Intriguingly, the putative endogenous TRPV4 channel activator (Earley and Brayden, 2015; Watanabe et al., 2003) 11,12-epoxyeicosatrienoic acid (11,12-EET, 1 µM) evoked currents in cECs even in the absence of dialyzed PIP_2_ (Figure 3—figure supplement 2). In any case, our results are the first to report PIP_2_-mediated suppression of TRPV4 channels in the endothelium and more broadly in native cells and are congruent with the results of Takahashi et al., 2014. Interestingly, a recent study provided structural evidence that lipid molecules are tightly bound to the selectivity filter of the TRPV4 channel pore, although the identity and function of these lipids were not characterized (Deng et al., 2018).

Canonical G_q_PCR signaling involves PLC activation and subsequent PIP_2_ breakdown into DAG and IP_3_. Receptor agonists that activate G_q_PCRs can dramatically lower PIP_2_ by promoting its hydrolysis at rates that exceed those of PIP_2_ re-synthesis. We show here that PGE_2_, which has previously been postulated to act as an NVC agent through actions on G_s_-coupled EP_2_/EP_4_ receptors in arteriolar smooth muscle (Lacroix et al., 2015; Zonta et al., 2003), activates TRPV4 channels in cECs independent of the action of the PIP_2_ metabolites, DAG and IP_3_, by relieving PIP_2_-mediated suppression (Figure 4, Figure 5). In contrast, we recently showed that G_q_PCR agonists exert the opposite effect on capillary Kir2.1 channels, inhibiting their ability to mediate capillary-to-arteriole electrical signaling (Harraz et al., 2018). In keeping with these previous findings and our current observations, simultaneous monitoring of TRPV4 and Kir2.1 channel currents confirmed the bidirectional effects of G_q_PCR agonists on the two channels (Figure 6). This two-way modulation (Figure 7) is unique as it indicates that a single G_q_PCR signaling cascade is capable of altering the balance between electrical Kir2.1 signaling (*inhibition*) and TRPV4 signaling (*facilitation*). Given the absence of Ca^2+^-activated K^+^ channels in cECs, noted above, G_q_PCR signaling-induced PIP_2_ depletion would likely depolarize cECs through the simultaneous disinhibition of depolarizing TRPV4 channels (this study) and deactivation of hyperpolarizing Kir2.1 channels (Harraz et al., 2018).

Based on our direct Kir2.1 current measurements (Harraz et al., 2018; Longden et al., 2017) and the known voltage- and K^+^-dependence of Kir2.1 channels (Longden and Nelson, 2015), we estimate that the outward current through these channels at physiological membrane potentials (about −40 mV) and external K^+^ (3 mM) is ~6 fA. Elevation of external K^+^ to 10 mM would increase Kir2 current at this voltage to ~260 fA. Though seemingly miniscule, such small membrane currents are precisely what is needed to ensure conduction fidelity. We have measured a sustained and profound hyperpolarization of about −25 mV in arteriolar smooth muscle in response to capillary stimulation with 10 mM K^+^ (Longden et al., 2017). Our computational modeling indicates that a stable membrane hyperpolarization of 25 mV requires the outward (hyperpolarizing) current to greatly exceed the inward (depolarizing) current. Our estimate of basal, PIP_2_-suppressed TRPV4 current at −40 mV is about −80 fA. G_q_PCR activation increases open probability ~6 fold, producing a current (400–500 fA) sufficient to effectively short circuit K^+^-induced hyperpolarization and cripple this key Kir2.1-based NVC mechanism. In conclusion, the low level of TRPV4 channel activity aligns with the functional role of capillaries.

Activation of capillary G_q_PCRs should also trigger Ca^2+^ signals in cECs, presumably through IP_3_-mediated Ca^2+^ release from intracellular stores as well as disinhibition of TRPV4 channels (as described here) and subsequent Ca^2+^ influx. Such capillary Ca^2+^ signals should positively influence hemodynamics—and thus functional hyperemia—providing a plausible explanation for the role of G_q_PCR agonists in the NVC process, presumably through the Ca^2+^-dependent activation of nitric oxide synthase (NOS) and subsequent release of the vasodilator, NO (Förstermann et al., 1991; Marziano et al., 2017). Capillary Ca^2+^ signaling might also serve an entirely different purpose in functional hyperemia: because local Ca^2+^ signals represent sites of PIP_2_ depletion (through G_q_PCR signaling), and thus membrane potential depolarization (through TRPV4 disinhibition and Kir2.1 deactivation), they are likely to interfere with the progression of electrical signals generated further down the vascular tree. Accordingly, these signals may represent ‘stop signs’ or ‘speed bumps’ that play a role in redirecting hyperpolarizing (vasodilatory) signals away from certain brain areas, in addition to their role in resetting the capillary membrane potential, noted above.

Given the role of PIP_2_ in negatively regulating TRPV4 channels, the exceedingly low basal TRPV4 activity and diminished sensitivity to GSK101 in cECs (Figure 1) compared with mesenteric artery ECs (Sonkusare et al., 2012) suggest differences in the PIP_2_ ‘set point’ in these two vascular beds. This supposition has important physiological implications for electrical signaling in the brain. Our data suggest that PIP_2_ levels in brain cECs are sufficient to saturate Kir2.1 channels (Harraz et al., 2018) and tune TRPV4 channel currents to levels that prevent inappropriate depolarization of the membrane potential (Figure 3). In the absence of PIP_2_ suppression of TRPV4 channel-mediated inward currents, it is unlikely that increased outward K^+^ currents through Kir2.1 channels in response to elevated K^+^ would be sufficient to cause membrane potential hyperpolarization. The mechanistic basis for the apparently higher PIP_2_ set point in brain cECs is currently unknown, but could include lower constitutive G_q_PCR activity, lower microenvironmental levels of G_q_PCR agonists and/or decreased G_q_PCR expression—and thus diminished PIP_2_ breakdown. Along these lines, a recent single-cell transcriptomics analysis showed that Gα_q_ (*Gnaq*) transcript levels in the mouse brain endothelium are reduced compared with those in peripheral pulmonary ECs (Vanlandewijck et al., 2018). Alternatively, differences in the set point could be indicative of more robust PIP_2_ synthesis in brain capillaries, a speculation that is supported by the higher mitochondrial content—and hence ATP synthesis—in highly active brain cECs compared with other ECs (Oldendorf and Brown, 1975; Oldendorf et al., 1977).

In conclusion, this study provides compelling evidence that brain capillaries express TRPV4 channels and further shows that the activity of these channels is physiologically suppressed by basal levels of PIP_2_. This introduces PIP_2_ and its modulation by G_q_PCR agonists as major regulators of brain capillary signaling. When maintained at sufficient levels, PIP_2_ inhibits TRPV4 channels and supports capillary-to-arteriole electrical signaling, but in response to G_q_PCR activation, PIP_2_ levels are reduced, enhancing TRPV4 signaling and inhibiting retrograde electrical signaling via Kir2.1 channels. Capillary G_q_PCRs can therefore be envisioned as molecular switches that dictate the signaling modality in the brain microvasculature.

## Materials and methods

**Key resources table:** 

Reagent type (species) or resource	Designation	Source or reference	Identifiers	Additional information
Strain, strain background (*Mus musculus*, males)	TRPV4 Knockout (TRPV4^-/-^) mice, C57BL/6J background	Thorneloe et al. (2008)	PMID: 18499743	
Strain, strain background (*Mus musculus*, males)	C57BL/6J	The Jackson Laboratory	RRID:IMSR_JAX:000664	
Chemical compound, drug	GSK1016790A (GSK101)	Sigma	Cat#: G0798	
Chemical compound, drug	Ruthenium red (RuR)	Sigma	Cat#: R2751	
Chemical compound, drug	Adenosine 5′-triphosphate magnesium salt (Mg-ATP)	Sigma	Cat#: A9187	
Chemical compound, drug	Adenosine 5′-triphosphate sodium salt	Sigma	Cat#: A2383	
Chemical compound, drug	HC-067047	Sigma	Cat#: SML0143	
Chemical compound, drug	KT5823	Sigma	Cat#: K1388	
Chemical compound, drug	PIK93	Tocris	Cat#: 6440	
Chemical compound, drug	Phenylarsine oxide (PAO)	Sigma	Cat# P3075	
Chemical compound, drug	H-89 dihydrochloride	Tocris	Cat# 2910	
Chemical compound, drug	Calphostin C	Tocris	Cat#: 1626	
Chemical compound, drug	LY294002 hydrochloride	Tocris	Cat#: 1130	
Chemical compound, drug	AH6809	Tocris	Cat#: 0671	
Chemical compound, drug	SC51322	Tocris	Cat#: 2791	
Chemical compound, drug	U-73122	Sigma	Cat#: U6756	
Chemical compound, drug	U-73343	Sigma	Cat#: U6881	
Chemical compound, drug	Poly-L-lysine hydrochloride	Sigma	Cat# 2658	
Chemical compound, drug	Gö6976	Calbiochem	Cat#: 365250	
Chemical compound, drug	Wortmannin	Sigma	Cat#: W1628	
Chemical compound, drug	PI(4,5)P2 (1,2-dioctanoyl) (sodium salt)	Cayman	Cat#: 64910	
Chemical compound, drug	PI(4,5)P2 (1,2-dipalmitoyl) (sodium salt)	Cayman	Cat#: 10008115	
Chemical compound, drug	Prostaglandin E_2_	Sigma	Cat#: 5640	
Chemical compound, drug	SLIGRL-NH2	Tocris	Cat#: 1468	
Chemical compound, drug	Carbachol	Sigma	Cat#: C4382	
Chemical compound, drug	Cyclopiazonic acid from *Penicillium cyclopium* (CPA)	Sigma	Cat#: C1530	
Chemical compound, drug	Guanosine 5′-triphosphate sodium salt (GTP)	Sigma	Cat#: G8877	
Chemical compound, drug	Adenosine 5′-[γ-thio]triphosphate tetralithium salt	Sigma	Cat#: A1388	
Chemical compound, drug	11,12-Epoxyeico satrienoic acid (11,12-EET)	Sigma	Cat#: E5641	
Chemical compound, drug	4α-Phorbol 12,13- didecanoate (4α-PDD)	Sigma	Cat#: P8014	
Chemical compound, drug	1,2-Bis (2-aminophenoxy) ethane-N,N,N′,N′- tetraacetic acid tetrapotassium salt (BAPTA)	Sigma	Cat#: A9801	
Software, algorithm	Prism	GraphPad	RRID:SCR_002798 https://www.graphpad.com/ scientific-software/prism/	
Software, algorithm	Clampfit 10.7	Axon Instruments	RRID:SCR_011323 https://www.moleculardevices.com/products/axon-patch-clamp-system	

### Animals

All procedures involving animals received prior approval from the University of Vermont Institutional Animal Care and Use Committee. Adult (2–3 month old) male C57BL/6J mice (Jackson Laboratories, USA) and TRPV4^-/-^ mice (Thorneloe et al., 2008) were group-housed on a 12 hr light:dark cycle with environmental enrichment and free access to food and water. Animals were euthanized by intraperitoneal injection of sodium pentobarbital (100 mg/kg) followed by rapid decapitation.

### Chemicals

1,2-Dioctanoyl phosphatidylinositol 4,5-bisphosphate sodium salt (diC8-PIP_2_) and 1,2-dipalmitoyl phosphatidylinositol 4,5-bisphosphate sodium salt (diC16-PIP_2_) were purchased from Cayman Chemical (USA). 12-(2-Cyanoethyl)−6,7,12,13-tetrahydro-13-methyl-5-oxo-5H-indolo(2,3-a)pyrrolo(3,4 c)-carbazole (Gö6976) was from Calbiochem (USA). *N*-((1*S*)−1-{[4-((2*S*)−2-{[(2,4-Dichlorophenyl) sulfonyl] amino}−3-hydroxy-propanoyl)−1-piperazinyl]carbonyl}−3-methylbutyl)−1-benzothiophene-2-carboxamide (GSK101) and 2-Methyl-1-[3-(4-morpholinyl)propyl]−5-phenyl-*N*-[3-(trifluoromethyl)phenyl]−1*H*–pyrrole-3-carboxamide (HC-067047) were obtained from Sigma-Aldrich (USA). 6-Isopropoxy-9-xanthone-2-carboxylic acid (AH6809), 8-chloro-2-[3-[(2-furanylmethyl)thio]−1-oxopropyl]-dibenz(*Z*)[*b*,*f*][1,4]oxazepine-10(11*H*)-carboxylic acid hydrazide (SC51322), *N*-[2-[[3-(4-bromophenyl)−2-propenyl]amino]ethyl]−5-isoquinolinesulfonamide dihydrochloride (H-89), (1*R*)−2-[12-[(2*R*)−2-(benzoyloxy)propyl]−3,10-dihydro-4,9-dihydroxy-2,6,7,11-tetramethoxy-3,10-dioxo-1-perylenyl]−1-methylethylcarbonic acid 4-hydroxyphenyl ester (calphostin C), and 2-(4-morpholinyl)−8-phenyl-4*H*-1-benzopyran-4-one hydrochloride (LY 294002) were purchased from Tocris (USA). Unless otherwise noted, all other chemicals were obtained from Sigma-Aldrich.

### Capillary endothelial cell isolation

Single ECs and capillary fragments were obtained from mouse brains by mechanical disruption of two 160 μm–thick brain slices using a Dounce homogenizer. Slices were homogenized in ice-cold artificial cerebrospinal fluid (124 mM NaCl, 3 mM KCl, 2 mM CaCl_2_, 2 mM MgCl_2_, 1.25 mM NaH_2_PO_4_, 26 mM NaHCO_3_, 4 mM glucose), and debris was removed by passing the homogenate through a 62 µm nylon mesh. Retained capillary fragments were eluted into dissociation solution composed of 55 mM NaCl, 80 mM Na-glutamate, 5.6 mM KCl, 2 mM MgCl_2_, 4 mM glucose and 10 mM HEPES (pH 7.3), containing neutral protease (0.5 mg/mL) and elastase (0.5 mg/mL) (Worthington, USA) plus 100 µM CaCl_2_, and incubated for 24 min at 37°C. Thereafter, 0.5 mg/ml collagenase type I (Worthington) was added and the sample was incubated for an additional 2 min at 37°C. The cell suspension was filtered and the residue was washed to remove enzymes. Single cells and small capillary fragments were dispersed by triturating 4–6 times with a fire-polished glass Pasteur pipette. Cells were stored in ice-cold isolation medium for use the same day (within ~6 hr).

### Electrophysiology

All patch-clamp electrophysiological recordings were performed at room temperature (∼22°C) in either the conventional or perforated whole-cell configuration. Currents were amplified using an Axopatch 200B amplifier, filtered at 1 kHz, digitized at 10 kHz, and stored on a computer for offline analysis with Clampfit 10.5 software. Recording pipettes were fabricated by pulling borosilicate glass (1.5 mm outer diameter, 1.17 mm inner diameter; Sutter Instruments, USA) using a Narishige puller. Pipettes were fire-polished to reach a tip resistance of ~4–6 MΩ. The bath solution consisted of 80 or 134 mM NaCl, 60 or 6 mM KCl, 1 mM MgCl_2_, 10 mM HEPES, 4 mM glucose, and 2 mM CaCl_2_ (pH 7.4). For the conventional whole-cell configuration, pipettes were backfilled with a solution consisting of 10 mM NaOH, 11.4 mM KOH, 128.6 mM KCl, 1.1 mM MgCl_2_, 2.2 mM CaCl_2_, 5 mM EGTA, and 10 mM HEPES (pH 7.2). A subset of experiments utilized a Mg^2+^-free solution, obtained by excluding MgCl_2_ from the pipette solution. In another group of cells, EGTA was replaced with BAPTA (5.4 mM). For perforated-patch electrophysiology, the pipette solution was composed of 10 mM NaCl, 26.6 mM KCl, 110 mM K^+^ aspartate, 1 mM MgCl_2_ and 10 mM HEPES; amphotericin B (200–250 µg/ml) was freshly added on the day of the experiment. Whole-cell capacitance, measured using the cancellation circuitry in the voltage-clamp amplifier, averaged 8.6 pF.

### Calculation of TRPV4 channel numbers

TRPV4 membrane currents were measured using the conventional whole-cell configuration and physiological concentrations of salts. Ruthenium red (RuR; 1 µM) was used to block Ca^2+^ influx and thereby prevent Ca^2+^ overload. We have shown previously that RuR causes a voltage-dependent block of TRPV channels and is rapidly driven out of the pore by membrane depolarization; at +100 mV, RuR (1 µM) blocks approximately 19% of the TRPV4 current (Sonkusare et al., 2012). The number of TRPV4 channels activated by 100 nM GSK101 was estimated by dividing the GSK101-induced current at +100 mV by the unitary current at this voltage (i.e. 10 pA) (Loukin et al., 2010; Watanabe et al., 2002) and correcting for the degree of RuR block.

### Data analysis

Data are expressed as means ± standard error of the mean (SEM). Effects of a given condition/treatment on whole-cell current were compared using paired or unpaired *t* tests or analysis of variance (ANOVA), as appropriate, using Graphpad Prism 7.01 software. p values≤0.05 were considered statistically significant. Additional patch-clamp data analyses were performed using Clampfit 10.5 software.

## Additional files

Transparent reporting form

### Data availability

All data generated or analyzed during this study are included in the manuscript and supporting source data files.

The following previously published datasets were used:

Vanlandewijck MHe LMäe MAndrae JBetsholtz C2017Single cell RNA-seq of mouse brain vascular transcriptomeshttps://www.ncbi.nlm.nih.gov/geo/query/acc.cgi?acc=GSE98816Publicly available at the NCBI Gene Expression Omnibus (accession no. GSE98816)

Vanlandewijck MHe LMäe MAndrae JBetsholtz C2017Single cell RNA-seq of mouse lung vascular transcriptomeshttps://www.ncbi.nlm.nih.gov/geo/query/acc.cgi?acc=GSE99235Publicly available at the NCBI Gene Expression Omnibus (accession no. GSE99235)
